# DNA methylation abnormalities induced by advanced maternal age in villi prime a high-risk state for spontaneous abortion

**DOI:** 10.1186/s13148-023-01432-w

**Published:** 2023-03-21

**Authors:** Meng Qin, Wei Chen, Lingyue Hua, Yan Meng, Jing Wang, Hanna Li, Rui Yang, Liying Yan, Jie Qiao

**Affiliations:** 1 Center for Reproductive Medicine, Department of Obstetrics and Gynecology, Peking University Third Hospital, Beijing, 100191 China; 2 National Clinical Research Center for Obstetrics and Gynecology (Peking University Third Hospital), Beijing, 100191 China; 3 Key Laboratory of Assisted Reproduction (Peking University), Ministry of Education, Beijing, 100191 China; 4 Beijing Key Laboratory of Reproductive Endocrinology and Assisted Reproductive Technology, Beijing, 100191 China; 5 Department of Obstetrics and Gynecology, Beijing Jishuitan Hospital, Beijing, 100096 China; 6 Department of Obstetrics and Gynecology, Peking University Third Hospital, Beijing, 100191 China; 7 National Center for Healthcare Quality Management in Obstetrics, Beijing, 100191 China; 8 Beijing Advanced Innovation Center for Genomics, Beijing, 100871 China; 9 Peking-Tsinghua Center for Life Sciences, Peking University, Beijing, 100871 China; 10 Research Units of Comprehensive Diagnosis and Treatment of Oocyte Maturation Arrest, Beijing Jishuitan Hospital, Beijing, 100191 China

**Keywords:** Advanced maternal age, Placenta, DNA methylation, Spontaneous abortion, GNE

## Abstract

**Background:**

Advanced maternal age (AMA) has increased in many high-income countries in recent decades. AMA is generally associated with a higher risk of various pregnancy complications, and the underlying molecular mechanisms are largely unknown. In the current study, we profiled the DNA methylome of 24 human chorionic villi samples (CVSs) from early pregnancies in AMA and young maternal age (YMA), 11 CVSs from early spontaneous abortion (SA) cases using reduced representation bisulfite sequencing (RRBS), and the transcriptome of 10 CVSs from AMA and YMA pregnancies with mRNA sequencing(mRNA-seq). Single-cell villous transcriptional atlas presented expression patterns of targeted AMA-/SA-related genes. Trophoblast cellular impairment was investigated through the knockdown of *GNE* expression in HTR8-S/Vneo cells.

**Results:**

AMA-induced local DNA methylation changes, defined as AMA-related differentially methylated regions (DMRs), may be derived from the abnormal expression of genes involved in DNA demethylation, such as *GADD45B*. These DNA methylation changes were significantly enriched in the processes involved in NOTCH signaling and extracellular matrix organization and were reflected in the transcriptional alterations in the corresponding biological processes and specific genes. Furthermore, the DNA methylation level of special AMA-related DMRs not only significantly changed in AMA but also showed more excessive defects in CVS from spontaneous abortion (SA), including four AMA-related DMRs whose nearby genes overlapped with AMA-related differentially expressed genes (DEGs) (*CDK11A*, *C19orf71*, *COL5A1,* and *GNE*). The decreased DNA methylation level of DMR near *GNE* was positively correlated with the downregulated expression of *GNE* in AMA. Single-cell atlas further revealed comparatively high expression of *GNE* in the trophoblast lineage, and knockdown of *GNE* in HTR8-S/Vneo cells significantly impaired cellular proliferation and migration.

**Conclusion:**

Our study provides valuable resources for investigating AMA-induced epigenetic abnormalities and provides new insights for explaining the increased risks of pregnancy complications in AMA pregnancies.

**Graphical Abstract:**

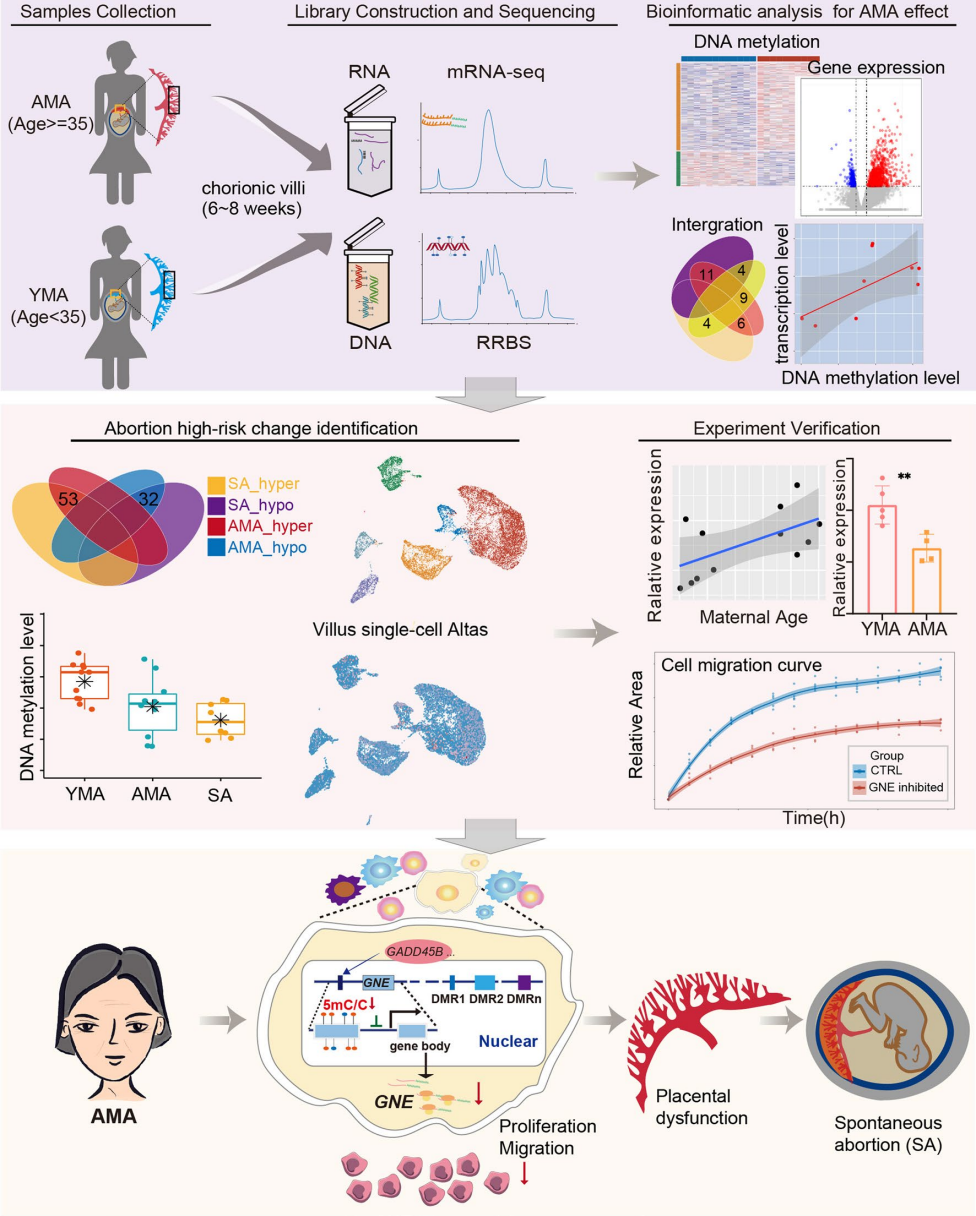

**Supplementary Information:**

The online version contains supplementary material available at 10.1186/s13148-023-01432-w

## Background

Advanced maternal age (AMA) refers to women giving birth at the age of 35 years or above [1]. The proportion of AMA in live births is rising in many developed and developing countries due to socioeconomic factors and increased choices available to women worldwide, such as the growing rate of advanced education, pursuit of a career, delayed marriage age, effective contraception, and application of assisted reproductive technology (ART) [2]. However, AMA has been proven to be associated with increased risks of many pregnancy complications [3, 4], especially disorders related to placental dysfunction, including miscarriage, preterm birth (PTB), preeclampsia (PE), and intrauterine growth restriction (IUGR) [5–9]. Previous studies have revealed abnormal morphological and cytological changes in AMA placentas. In humans, AMA placentas were reported to have larger weights and more relaxed uterine arteries [10]. Defective differentiation of trophoblast cells [11], stunted uterine artery remodeling [12], and increased levels of apoptosis and oxidative stress [13] have also been observed in the placentas of AMA mice and rat models. Although it has been suggested that the decreased expression of specific genes, such as *α-Klotho* [14] and *SIRT1* [15], might play a role in premature placental senescence in AMA pregnancies, the molecular mechanism behind this phenomenon remains largely unexplored.

Epigenomes play important roles in regulating gene expression [16]. As an important modifiable epigenetic mechanism, abnormal DNA methylation is closely associated with the occurrence of various diseases [17] and is implicated in interfering with placental development and function [18]. For example, altered DNA methylation in the promoters of *SERPINA3*, *APC,* and *RASSF1A* genes has been reported in the placentas of patients with PE [19]. Decreased methylation levels in the imprinted controlled region (ICR) of *H19*/*IGF2* have also been commonly observed in IUGR placentas [20, 21]. In addition, specific DNA methylation patterns show high variability during the process of aging, and the combined DNA methylation pattern in some CpG sites has been widely identified as the “epigenetic clock,” which could reflect the biological age during the human lifespan [22]. In fact, multiple studies have suggested that abnormal DNA methylation plays an important role in aging-related diseases, such as Alzheimer’s disease, type 2 diabetes, and various cancers [23].

The reproductive system is affected by the biologically unavoidable fate of aging, leading to a gradual decline in female fecundability that begins in the late 20 s but accelerates after the mid-30 s [24]. Consistent with this phenomenon, it has been widely reported that AMA induces aneuploidies, mitochondrial dysfunction, and transcriptional alterations in human oocytes [25–27]. AMA mouse modeling has further revealed reduced expression in oocytes for genes involved in the establishment and maintenance of DNA methylation, such as *Dnmt1*, *Dnmt3a*, *Dnmt3b,* and *Dnmt3L* [28], along with lower genome-wide DNA methylation in both oocytes and following embryos [29, 30]. Meanwhile, with increased maternal age, maternal age-dependent changes have also been observed in the local DNA methylome of human ovarian granulosa cells [31, 32], in the specific CpG sites of umbilical cord blood, and even in the specific CpG sites of the peripheral blood of adult daughters [33–35]. Thus, relatively older maternal age in AMA pregnancies might also cause abnormal DNA methylation in the placenta and serve as a potential key factor for the increased risk of placenta-associated diseases in AMA.

However, the epigenetic influence of AMA on the placenta has long been neglected. Only one study in mice reported by Paczkowski et al. has tried to explore AMA’s influence on the placenta, reporting DNA methylation alterations at several ICRs (*CDKN1C*, *GNAS*, *IGF2*, etc.) [30]. The DNA methylation pattern of the human placenta during AMA pregnancy is still blank, and a comprehensive genome-wide investigation of the influence of AMA on the DNA methylome is urgently needed. Thus, we applied RRBS and mRNA-seq to conduct a combined analysis of both the DNA methylome and transcriptome on the induced chorionic villi samples (CVSs) from induced abortion cases in both AMA and young maternal age (YMA) pregnancies as well as the CVSs from SA cases. Our results revealed that AMA induced local, rather than global, alterations in the villous DNA methylome, a part of which might be closely correlated with SA. It deepens our understanding of the influences of AMA and provides novel evidence to develop strategies for the diagnosis, providence and even therapy for pregnancy complications in AMA.

## Results

### The DNA methylation level in the global genome and specific elements

**Fig. 1:**
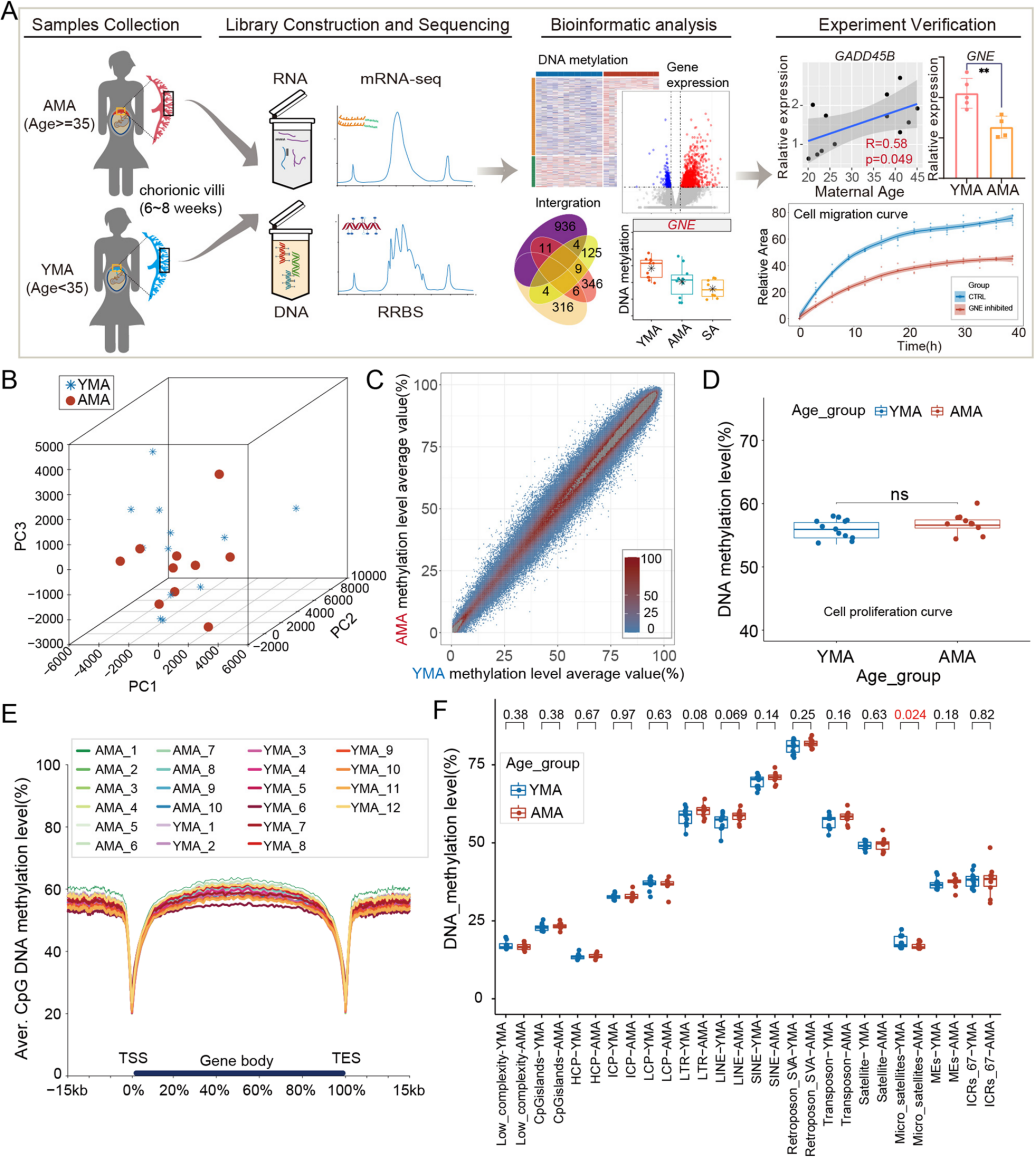
DNA methylation level in the global genome and specific elements. **A** Flowchart of the experiment and data analysis. **B** Three-dimensional scatter plot of PCA showed the distribution of CVSs based on the DNA methylation pattern of all 200 bp bins (sample size: *n* = 22 and bin number: *n* = 684,403). The first three principal components of PCA were applied. **C** Scatter plot showed the distribution of the average DNA methylation value of each bin in both the AMA group and YMA group. The X-axis indicates the average methylation level of the AMA group, and the Y-axis denotes the average methylation level of the YMA group. Pearson correlation analysis was performed to calculate the correlation between the two groups (R = 1, *P* < 0.01). **D** Box and dot plot shows the distribution of the average DNA methylation levels of each sample. The P value between the two groups was determined using the Wilcoxon rank-sum test (*P* = 0.23). **E** Line charts presented the average DNA methylation level along the gene body, as well as 15 kilobases (kb) upstream of the transcription start site (TSS) and 15 kb downstream of the transcription end site (TES) of all genes. Each color refers to a specific sample. **F** Box and dot plot shows the distribution of the average DNA methylation levels of each sample for specific genome elements. P values between the two groups were determined using the Wilcoxon rank-sum test

All CVSs were obtained in the first trimester of pregnancy (6–8 weeks of gestation, Additional file 13: Table S1) and were divided into AMA group (> 38 years, with a mean age of 40.33 ± 1.50, *n* = 12) and YMA group (< 30 years, with a mean age of 22.75 ± 2.30, *n* = 12) according to maternal age (Fig. 1A). Given the high incidence of chromosomal copy number variations (CNVs) in AMA pregnancies, we conducted CNV analysis of the samples after data quality assessment and excluded all samples with CNVs (Additional file 1: Figure S1A and Additional file 14: Table S2). After splitting the genome into 200 bp bins, only those containing no fewer than three CpG sites and existing in more than 80% of the samples were used for downstream analysis (Additional file 1: Figure S1B). Finally, a total of 684,403 bins in 12 samples from the YMA group and 10 samples from the AMA group were included in the analysis (Additional file 1: Figure S1B).

Both unsupervised hierarchical clustering analysis and principal component analysis (PCA) based on the DNA methylome showed no obvious isolation between the AMA and YMA groups (Additional file 2: Figure S2A and Fig. 1B). The global distribution of the methylation levels of bins was similar among samples, as well as between the AMA and YMA groups (Additional file 2: Figure S2B and Fig. 1C). The average methylation level in the AMA group was higher than that in the YMA group, but the difference between the two groups did not reach statistical significance (P value = 0.23, Wilcoxon rank-sum test) (Fig. 1D). The overall DNA methylation level around the gene body was slightly higher in the AMA group than in the YMA group. (Figs. 1E and Additional file 2: Figure S2C). Except for microsatellites, there were no significant intergroup differences in the methylation levels of specific genomic elements (Fig. 1F). These results indicate that AMA does not induce dramatic global changes in the CVS DNA methylome.

### AMA-related local alteration of the DNA methylome

**Fig. 2:**
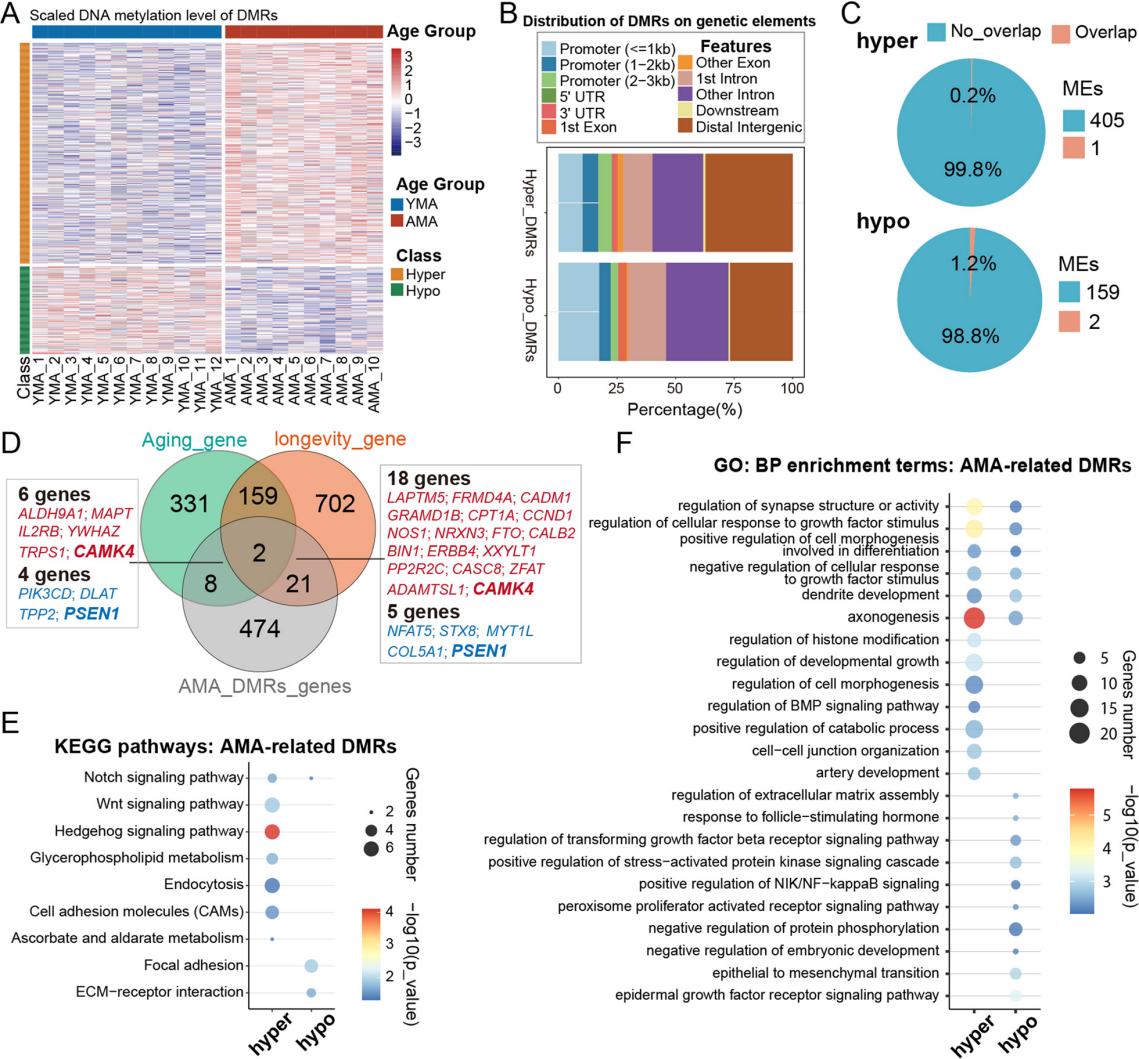
Potential influence of AMA-induced local DNA methylation alteration. **A** Heatmap shows the scaled methylation level of each sample in hyper- or hypo-AMA-related DMR regions. **B** Column graph shows the percentage of AMA-related DMRs in different genetic elements. Hyper-DMR is on the top, while hypo-AMA-related DMR is on the bottom. **C** Pie charts show the proportion of hyper- or hypo-AMA-related DMRs located in or outside the ME regions. **D** Venn diagram presents the relationships among the nearest genes of AMA-related DMRs, genes in the longevity database, and genes recorded in the Aging Atlas database. Overlapping genes near AMA-related DMRs are shown in the frame. Genes associated with hyper-AMA-related DMRs are marked in red, while genes associated with hypo-DMRs are marked in blue. The bolded gene symbols refer to overlapping genes existing in both the longevity database and the Aging Atlas database. **E** Bubble chart shows representative KEGG pathways enriched for AMA-related DMR. The P value was determined by a hypergeometric test. **F** Bubble chart shows representative biological process Gene Ontology (GO) terms enriched for AMA-related DMR. The P value was determined using a hypergeometric test

To further investigate the influence of AMA on the DNA methylome of CVS, we performed an intergroup comparison for the DNA methylation level of each bin, which revealed that the vast majority of difference values were distributed between -15% and 15% (Additional file 3: Figure S3A, 99.92% of 200 bp bins). In total, 566 differentially methylated regions (DMRs) were identified between the AMA and YMA groups (| df |≥ 15% and *q* value < 0.05; hypo-AMA-DMRs: *n* = 160; hyper-DMRs: *n* = 406; hypo-*vs.* hyper: 28.4% *vs.* 71.6%) (Fig. 2A, Additional file 3: Figure S3B-D; see Additional file 15: Table S3 for details). AMA-related DMRs were distributed on all chromosomes, with only approximately 25% located in promoter regions (Fig. 2B and Additional file 3: Figure S3E). Functional genomic annotation also revealed that quite a few AMA-related DMRs were located in repeated elements, including SINE (Hyper: 34.5% and Hypo: 43.5%), LINE (Hyper: 17.2% and Hypo: 13%), and LTR (13.5%–14.9%) (Additional file 3: Figure S3F). Meanwhile, one hyper-DMR and two hypo-DMRs were directly located in metastable epiallele (ME) regions [36] (Fig. 2C), of which the DNA methylation pattern is randomly established in embryos and then stably maintained in differentiated tissues and affected by the early pregnancy environment [37].

Next, we searched the nearest genes of AMA-related DMRs in the LongevityMap and Aging Atlas database [38, 39] and found that 31 genes had been reported to be associated with either aging or longevity. Among them, 23 genes (*CAMK4*, etc.) were hypermethylated, whereas eight genes (*PSEN1*, etc.) were hypomethylated in the AMA group (Fig. 2D). To further investigate the specific biological implications of AMA-related DMRs, we conducted Gene Ontology (GO) enrichment analysis for the nearest genes of AMA-related DMR and found that these genes were enriched in the processes involved in epithelial-to-mesenchymal transition, regulation of extracellular matrix assembly, and artery development (Fig. 2E; see Additional file 16: Table S4 for details), which are essential for placental development [40]. Kyoto Encyclopedia of Genes and Genomes (KEGG) pathway enrichment analysis revealed that WNT pathways, NOTCH signaling pathways, focal adhesion, and extracellular matrix (ECM)-receptor interaction were the representatively enriched pathways (Fig. 2F). Together, these results suggest that AMA may induce local DNA methylation changes in functional genomic regions that are associated with aging-like phenotypes and are involved in important biological processes for placental development.

### Abnormal change in gene expression in the AMA group

**Fig. 3:**
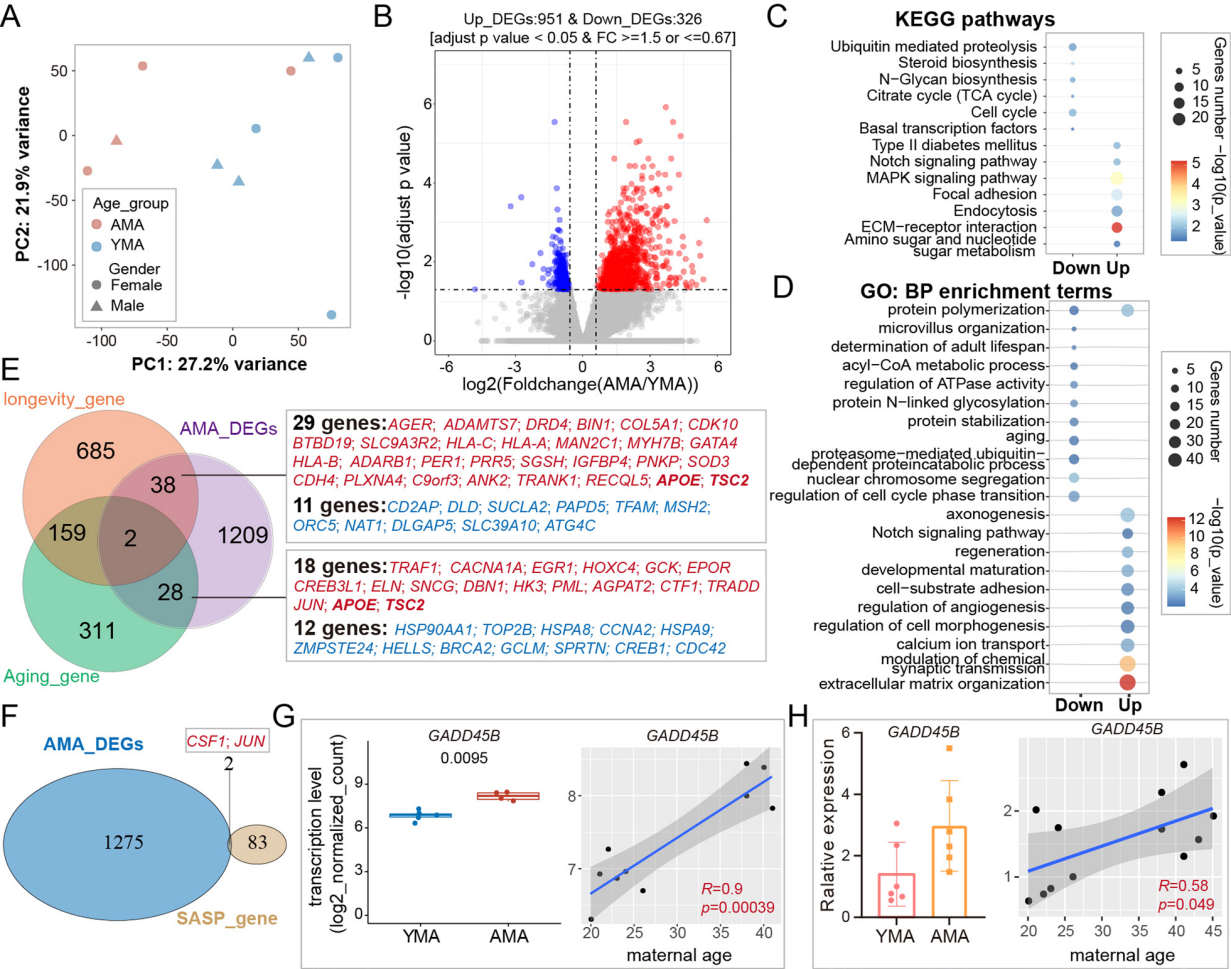
AMA-related changes in the transcriptome of CVSs. **A** Two-dimensional scatter plot of PCA shows the distribution of all samples (sample size: *n* = 20); the first two principal components of PCA based on global gene expression were used. **B** Volcano plot shows AMA-related DEGs identified between the AMA and YMA groups, with upregulated DEGs marked as red spots and downregulated DEGs marked as blue spots. **C** Bubble chart shows the representative KEGG pathways for AMA-related DEGs. The *P* value was determined using a hypergeometric test. **D** Bubble chart showed the representative biological process GO terms enriched for AMA-related DEGs. The P value was determined using a hypergeometric test. **E** Venn diagram presents the relationship between AMA-related DEGs and genes recorded in either the longevity database or the Aging Atlas database. Overlapping genes near AMA-related DMRs are shown in the frame. Upregulated DEGs are marked in red, while downregulated DEGs are marked in blue. The bolded gene symbol refers to overlapped genes existing in both longevity database and Aging Atlas database. **F** Venn diagram shows the relationship between AMA-related DEGs and SASP genes. **G** Box and dot plot (left) shows the gene expression level of *GADD45B* in the AMA (red) and YMA (blue) groups; the P value between the two groups was determined using the Wilcoxon rank-sum test. Scatter diagram (right) shows the gene expression level of *GADD45B* with maternal age. The fitted linear regression line is shown in blue. **H** Column diagram (left) displays the relative gene expression level of *GADD45B* determined by qRT-PCR in the AMA (orange) and YMA (pink) groups. Each dot represents the gene expression level of a detected sample. Error bars represent the standard deviation. Scatter diagram (right) shows the relative gene expression level of *GADD45B* with maternal age. The fitted linear regression line is shown in blue. The correlation coefficient and P value between maternal age and *GADD45B* expression level in **G** and **H** were both calculated using Spearman correlation tests

To explore the transcriptional effect of AMA-related DMRs and candidate upstream regulatory factors, we performed mRNA-seq on CVSs collected from the AMA and YMA groups (Additional file 13: Table S1 and Additional file 4: Figure S4A). PCA and unsupervised hierarchical clustering analysis based on gene expression revealed that samples in the AMA group tended to separate from those in the YMA group (Fig. 3A and Additional file 4: Figure S4B-4C). A total of 1277 differentially expressed genes (DEGs, fold change ≥ 1.5 or  ≤ 0.67, adjusted P value < 0.05) were identified between the two groups (upregulated AMA-related DEG: *n* = 951; downregulated AMA-related DEG: *n* = 326) (Fig. 3B and Additional file 4: Figure S4D; see Additional file 17: Table S5 for details). Notably, KEGG pathway enrichment analysis showed that DEGs were highly enriched in the NOTCH signaling pathway, focal adhesion, and ECM–receptor interaction (Fig. 3C; see Additional file 18: Table S6 for details), which were also mentioned in the enrichment analysis for DMRs (Fig. 2F). GO analysis of DEGs showed the enrichment of extracellular matrix organization, cell–substrate adhesion, as well as microvillus organization, and also underlined AMA-related transcriptional disturbance in the aging and regeneration processes (Fig. 3D; see Additional file 18: Table S6 for details). Consistently, we found that 68 DEGs have already been reported in the longevity and aging databases (Fig. 3E). Meanwhile, it was observed that the typical senescence-associated secretory phenotype (SASP) genes [41], *JUN* and *SCF1*, were significantly upregulated in the AMA group (Fig. 3F).

Notably, several factors involved in DNA demethylation showed significant differences between the AMA and YMA groups, including *GADD45A*, *GADD45B, XRCC*, *MBD4*, and *TDG*, which were accompanied by a high correlation coefficient with maternal age (Fig. 3G and Additional file 4: S4E-4F). Quantitative reverse transcription polymerase chain reaction (qRT-PCR) further confirmed the upregulated tendency of *GADD45B* in the AMA group and its high correlation with aging (Fig. 3H) *GADD45B* has been reported to take part in the DNA demethylation process by recruiting and acting as scaffolds for cytidine [42]. This finding implies that the local alteration of DNA methylation might originate from the disorder of these regulators during AMA pregnancy. In general, the above results suggest that AMA-induced alterations related to aging-like effects and placental dysfunction are not limited to the DNA methylome, but also extend to the transcriptome.

### Specific transcriptional changes correlated with DNA methylome alterations

**Fig. 4:**
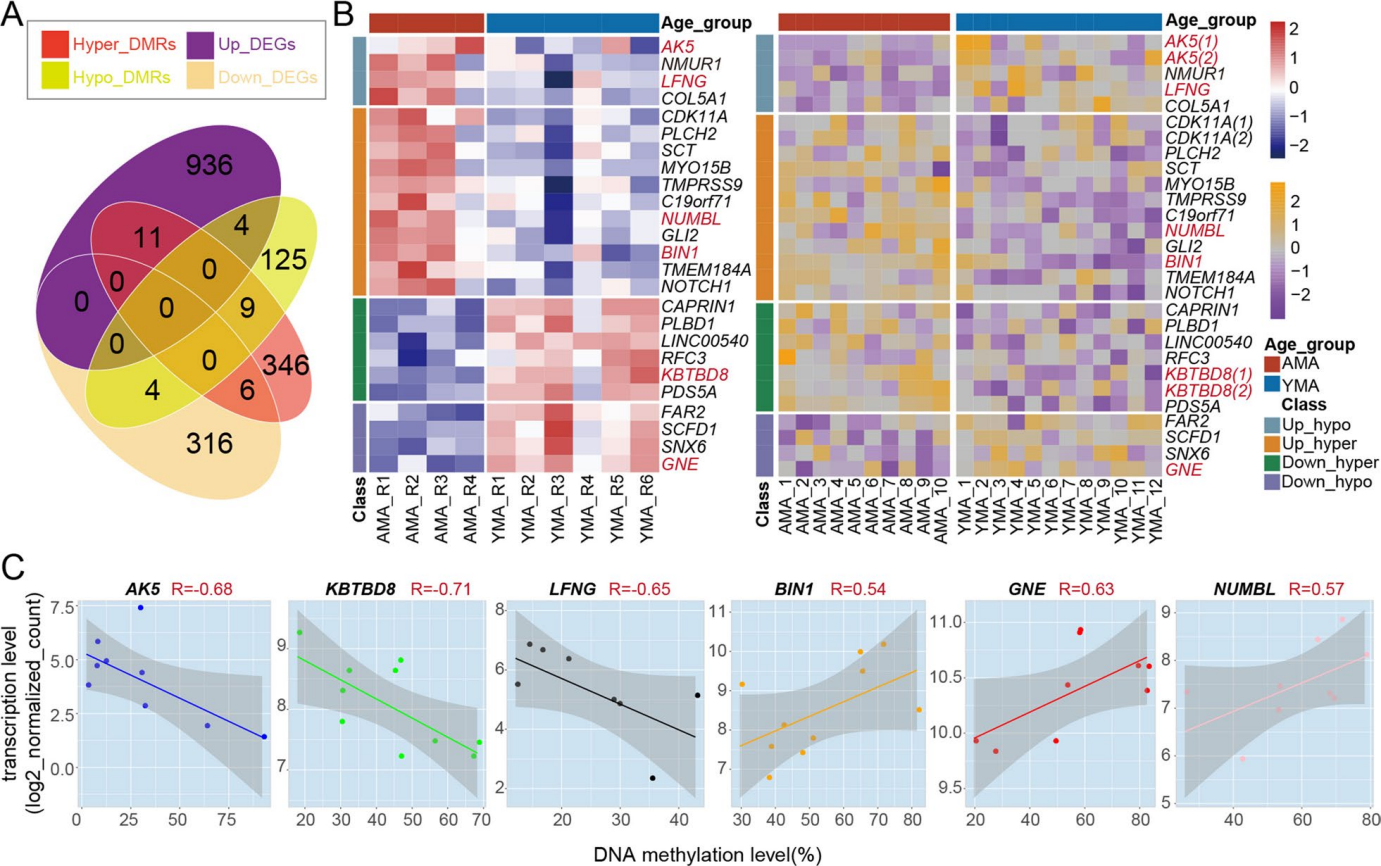
Integrated analysis of AMA-related changes in both DNA methylome and transcriptome. **A** Venn diagram displays the relationship between AMA-related DEGs and the nearest genes of AMA-related DMRs. **B** Heatmaps present the scaled gene expression level (left) of AMA-related DEGs and scaled DNA methylation level (right) of DMRs in each sample. The gene symbols of DEGs or the nearest genes of AMA-related DMRs identified in (**A**) are shown to the right of the heatmap, respectively. **C** Scatter diagram shows the correlation between the gene expression level and the DNA methylation level of the nearby DMRs of six target AMA-related DEGs (*AK5, GNE, KBTBD8, NUMBL, BIN1, KBTBD8*, and *LFNG*). The straight line refers to the fitted linear regression line. The correlation coefficient and P value were calculated using Spearman-based correlation tests, and the shaded areas represent 95% confidence intervals around each slope

To further explore the specific relationship between AMA-induced changes in the DNA methylome and the transcriptome, we performed an integrated analysis of DMRs and DEGs. There were 25 genes in total changed in both gene expression and the DNA methylation level of nearby DMRs: four upregulated DEGs and four downregulated DEGs overlapped with the nearest genes of hypo-DMRs, while 11 upregulated DEGs and six downregulated DEGs overlapped with the nearest genes of hyper-DMRs (Fig. 4A, B and Additional files 5, 6, 7: Figure S5-S7; see Additional file 19: Table S7 for detail). We further calculated the correlation coefficient between the DNA methylation level and the gene expression level of these 25 target genes using paired samples (Additional file 8: Figure S8A) and identified six DEGs with a relatively high correlation (|R|> 0.5; *AK5, GNE, KBTBD8, NUMBL, BIN1,* and *LFNG*) (Fig. 4C and Additional file 8: Figure S8A-8B). Meanwhile, both the expression levels of these genes and the DNA methylation levels of their nearby DMRs showed a high correlation with maternal age (|R|> 0.5) (Additional file 8: Figure S8C). qRT-PCR of CVSs in the expanded cohort confirmed the alteration trends in the expression of five genes (Additional file 8: Figure S8D). Overall, it suggests that AMA-induced local DNA methylation alterations are in relatively weak association with transcriptional alterations, but the changes in specific locations may directly disturb the transcriptional levels of the corresponding genes.

### AMA-induced abnormal DNA methylation changes associated with spontaneous abortion and cellular dysfunction

**Fig. 5:**
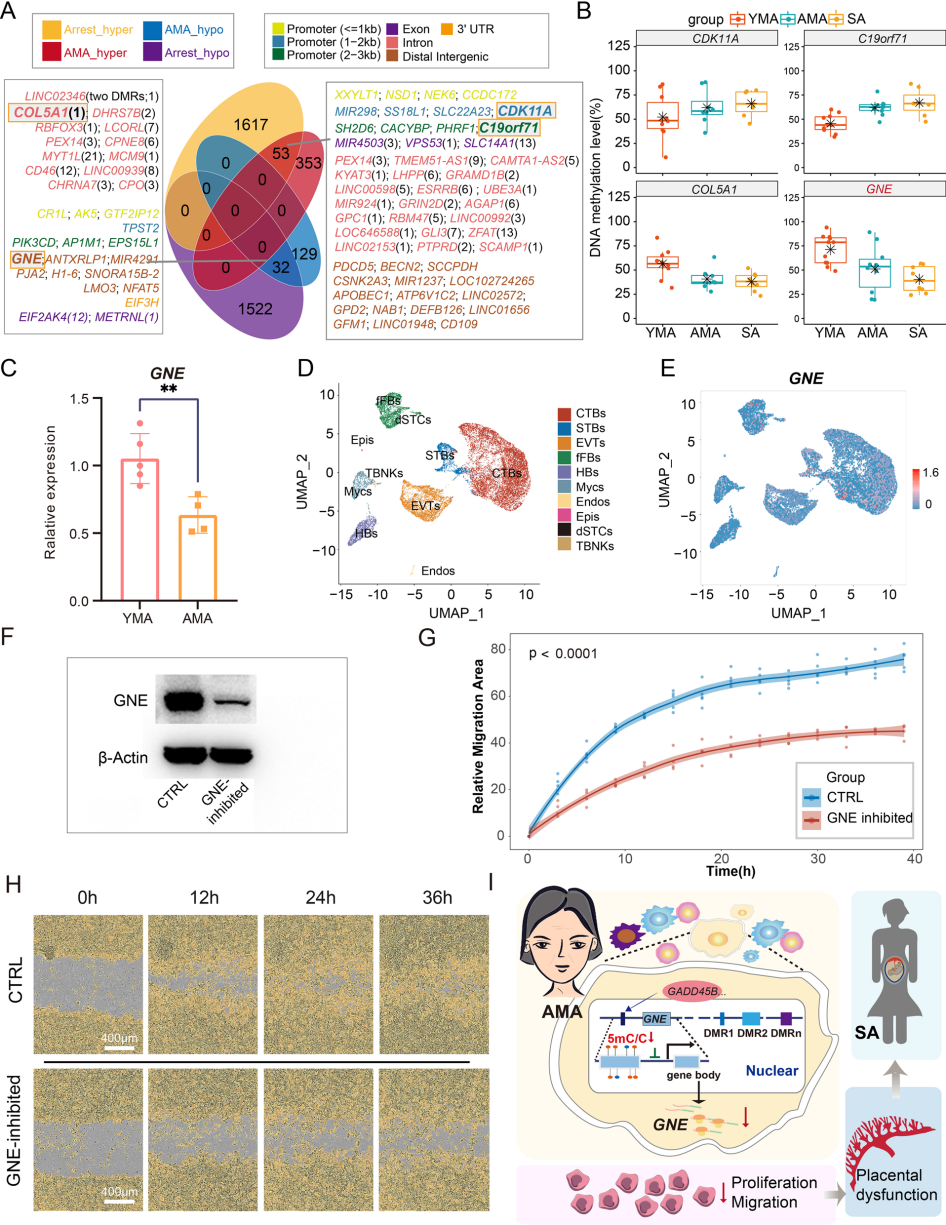
Trophoblast dysfunction associated with the DNA methylation change in the DMR near the *GNE* gene in both AMA and SA.** A** Venn diagram shows the relationship between AMA-related DMRs and SA-DMRs. Genes near the overlapping DMRs are shown in the frame. The different colors of gene symbols refer to the different genetic elements where DMRs are located. The numeral suffix after a gene symbol refers to the serial number for a specific exon or intron in the reference gene. The bolded gene symbols refer to the four genes near the DMRs shown in **B**. **B** Box and dot plot show the distribution of DNA methylation levels of four DMRs near *CDK11A*, *C19orf71*, *COL5A1*, and *GNE* in the AMA, YMA, and SA groups. The changes between SA and YMA were larger than those between AMA and YMA in these four DMRs. **C** Column diagram shows the relative gene expression level of *GNE* determined by qRT-PCR in the AMA (orange) and YMA (pink) groups. Each dot represents the gene expression level of a detected sample. The P value was determined using an unpaired t test (**P < 0.01). **D** UMAP visualization presents the single-cell cluster*s* of human villus cells reconstructed using the first-trimester placental data [43]. Colors indicate cell types. CTBs, cytotrophoblasts; STBs, syncytiotrophoblasts; EVTs, extravillous trophoblasts; fFBs, fetal fibroblasts; HB, Hofbauer cells; Mycs, maternal macrophages; Endo, endothelial cells; Epi, epithelial cells; dSTCs, decidual stromal cells; TBNKs, the mixed population consisting of T cells, B cells, and natural killer cells. **E** UMAP visualization displays the expression pattern of *GNE*. **F** Western blot analysis of GNE expression levels in HTR8-S/Vneo cells treated with *GNE* siRNA (15 pmol/L). **G** Point graph shows the relative migration area in different time points for both the *GNE* inhibition group (red) and control group (CTRL, blue). The lines refer to the fitting curves. The P value between the AMA and YMA groups was determined using two-way ANOVA (*P* < 0.0001). **H** Cell migration assays for HTR8-S/Vneo cells in representative time points (0 h, 12 h, 24 h, 36 h) for CTRL (top) and *GNE* siRNA (bottom) groups. **I** A schematic illustration presents the close connection between advanced maternal age and placental dysfunction. AMA-induced abnormal alterations in specific local DNA methylation might disturb the gene expression of corresponding genes (*GNE*, etc.), which will reduce the cellular capability of villous cells and thus lead to the increased risk of pregnancy disorders, such as spontaneous abortion

To investigate the underlying relationship between AMA-induced changes in the DNA methylome and adverse pregnancy outcomes in AMA, we further obtained the DNA methylome of CVS from spontaneous abortion (SA) patients (27.55 ± 2.77; embryo gender: female, *n* = 5; male, *n* = 7) and identified 2167 SA-related DMRs (hyper-DMRs: *n* = 1670; hypo-DMRs: *n* = 1554) by comparing eight samples without CNVs in the SA group with those in the YMA group (Additional file 9: Figure S9; see Additional file 20: Table S8 for details). The integrated analysis between SA-related and AMA-related DMRs revealed 53 hyper-DMRs and 32 hypo-DMRs with the same tendency, whereas no DMRs showed the opposite tendency (Fig. 5A). In particular, we also investigated the DNA methylation level of AMA-related DMRs corresponding to the 25 overlapping genes mentioned previously (see Fig. 4B) and found that changes between SA and YMA in four DMRs (nearest genes or related DEGs: *CDK11A*, *C19orf71*, *COL5A1*, and *GNE*) were not just significantly different but even larger than those between AMA and YMA (Fig. 5B, Additional files 5, 6: Figure S5-S6, and Additional file 10: Figure S10A-10B). This implies that some AMA-induced local changes in the DNA methylome, along with closely related transcriptional fluctuations, may partly account for the high risk of spontaneous abortion in AMA pregnancies.

Consistent with the high possibility of directly regulating the gene expression for AMA-related DMRs near *GNE*, as shown in Fig. 4C, qRT-PCR confirmed the significant downregulation of *GNE* in the AMA group compared with the YMA group (Fig. 5C and Additional file 8: Figure S8D). Moreover, we reanalyzed the single-cell transcriptional atlas of early CVS [43] (Fig. 5D) and found that *GNE* was mainly expressed in the trophoblast cell lineage, suggesting its key role in trophoblast cells (Fig. 5E and Additional file 11: Figure S11). Knockdown of the *GNE* gene in HTR8-S/Vneo cells led to a reduced capacity for migration and proliferation, but not invasion (Fig. 5F–H and Additional file 12: Figure S12). Altogether, the above results indicated that excessive disturbance of DNA methylation at specific DMRs is associated with an increased incidence of spontaneous abortion due to the dysfunction of placental cells (Fig. 5I).

## Discussion

AMA is regarded as an independent risk factor for many pregnancy complications [4]. In this study, we revealed that AMA-induced local DNA methylation changes were involved in the process of aging and cell adhesion in early CVS, which was also reflected in the specific alterations in the transcriptome. Meanwhile, larger immoderate DNA methylome changes were observed in specific genomic regions of CVS from SA. The abnormally decreased expression of *GNE*, highly correlated with the decreased DNA methylation level of the nearby DMR, damaged the migration and proliferation capabilities of trophoblast cells. AMA-related DNA methylation abnormalities, damaging the gene expression regulation network and villus cellular function, could partly explain the increased risk of pregnancy complications in AMA such as SA.

Although there were no significant differences, the average methylation level in CVS was higher, with a large percentage of DMRs (71.6%) being hypermethylated in AMA than in YMA. However, abundant studies on the influence of aging on DNA methylation revealed that overall DNA methylation levels decreased with increasing age in the blood and some tissues [44]. Four CpG sites on the *KLHL35* gene and 135 of 144 CpG sites in cord blood have been reported to present a decreased methylation level with increasing maternal age [34, 35]. Further, another epigenome-wide association study (EWAS) of the peripheral blood of 2,740 adult daughters revealed that 84% (*n* = 73) of maternal age-related CpG sites displayed lower DNA methylation levels [33]. One closely related theory suggests a notable pattern for DNA methylation change during the aging process, in which the originally low-methylation genomic region tends to increase the methylation level [44, 45], whereas the originally high-methylated region tends to lose methylation. This theory might account for the increased global DNA methylation levels observed in AMA-CVS due to the intrinsically lower average DNA methylation level of the placenta than in most somatic tissues [46]. In addition to the decreased methylation pattern of specific CpG sites along with increased maternal age in offspring, our recent work directly comparing the DNA methylome of cord blood between YMA and AMA also uncovered a slightly lower average genome-wide DNA methylation level and more hypo-DMRs in AMA [47]. Thus, the AMA-related DMRs identified in early CVS further support the view that the epigenomes of both intra- and extra-embryonic tissues are disturbed during AMA pregnancy.

Quite a few AMA-related DMRs were located in repeated elements, including SINE, LINE, and LTR, which corresponded to results of a previous report of increased variability in the DNA methylation status of repeat elements, such as Alu and LINE-1, during the aging process [44] and suggested an aging-like feature in the CVS of AMA. Our results also revealed that three AMA-related DMRs were directly located in the ME regions. Since the patterns in MEs are established during the preimplantation embryos and are sensitive to the disturbance of genetic and peri-conceptional environments, changes in these regions are generally conserved across multiple tissues [37]. It is again reasonable to assume that AMA-induced changes in part of those DMRs might occur in the early embryos and therefore were not limited to the CVS but also systemically existed among multiple tissues in the offspring. In addition, genes near AMA-related DMRs were highly enriched in the WNT and NOTCH signaling pathways. Canonical WNT signaling controls CTB progenitor expansion and regulates the migration and differentiation of EVTs, whereas NOTCH1 signaling controls EVT progenitor development and promotes their growth and survival [48]. Another enriched process, the TGF-β signaling pathway, has also been reported to regulate EVT invasion [49]. Taken together, these results suggest that AMA-induced DNA methylation changes have an extensive influence on the development of the human placenta.

AMA also causes a series of transcriptional abnormalities in CVS. The expression levels of six AMA-related DEGs (*AK5*, *GNE*, *KBTBD8*, *NUMBL*, *BIN1*, and *LFNG*) may be directly regulated by DNA methylation of nearby DMRs. Although only a few AMA-related DEGs belonged to the nearest genes of AMA-related DMRs, there were many co-affected biological processes and pathways involved in trophoblast cell function, such as processes associated with axon genesis, extracellular matrix organization, and regulation of cell morphogenesis, as well as pathways related to NOTCH signaling, ECM–receptor interaction, endocytosis, and focal adhesion. *NUMBL* and *LFNG,* two of the six AMA-related DEGs mentioned above, were involved in axon genesis and the NOTCH signaling pathway, respectively. Meanwhile, the few overlaps between AMA-related DEGs and genes nearby DMRs may suggest other mechanisms in the placental gene regulation besides DNA methylation. It is well known that the special gene regulation mechanisms exist in placental development and H3K27me3 is emphasized to play a very important role in regulating the expression of genes which are generally controlled by DNA methylation in other somatic tissues such as ICRs [50, 51]. Importantly, premature placental senescence has been observed in both human and mouse models, and senescence markers such as p53, p21, and p16 were significantly upregulated in both human term placentas and first-trimester placentas [14]. In accordance with this research, our study also revealed many AMA-related DEGs, and the nearest genes of AMA-related DMRs were reported in the aging/longevity database or involved in the biological processes of aging, suggesting aging-like features in the placenta during AMA pregnancy.

We also found transcriptional abnormalities in AMA for five genes (*GADD45A*, *GADD45B*, *XRCC1*, *MBD4,* and *TDG*) that are involved in DNA demethylation through the base excision repair (BER) pathway [52]. A decline in *TDG* and DNA demethylase *TET1/3* has been reported during the aging process in human peripheral blood mononuclear cells [53]. Overexpression of *TET3*, repression of *TDG* [54], and downregulation of DNA methyltransferases (Dnmt1, Dnmt3a, Dnmt3b, and Dnmt3L) [29] were also observed in MII oocytes from aging mice. *GADD45* proteins have been reported to be highly associated with aging and longevity [55, 56]. Gadd45a inhibits mammary tumor growth through p38-mediated cellular senescence [55, 56]. Although Gadd45b is regarded as a senescence-associated marker [57, 58], knockout of Gadd45b caused premature liver senescence and opposed the promotion of senescence by Gadd45a in a mouse model [55, 56]. Thus, these observations revealed a close relationship between the DNA methylation changes and the adverse expression alteration of these DNA methylation regulators in AMA, but the detailed regulatory mechanism needs further exploration in future research.

Previous studies have reported altered DNA methylation of *FOXP3*, *CREB5*, *H19*, *LIT1,* and *SNRPN* in SA [59–61], suggesting that abnormal DNA methylation of specific genes could contribute to human pregnancy loss. Importantly, our study revealed that the DNA methylation changes in many AMA-related DMRs were extremely dramatic in SA and even exceeded the AMA-induced methylation changes, including four DMRs whose nearby genes overlapped with the AMA-related DEGs (*CDK11A*, *C19orf71*, *COL5A1,* and *GNE*). The *C19orf71* gene has been reported to be associated with skin pigmentation [62] and occupational attainment [63] in genome-wide association studies (GWAS). C19orf71, also named tektin bundle interacting protein 1 (TEKIP1), is observed in the center of the tektin bundle in bovines and is suspected to recruit tektins or for the stabilization of the bundle and may thus take part in the regulation of ciliary motility [64]. Thus, aberrant DNA methylation in special AMA-related DMRs in CVSs might partly explain the increased risk of SA and other pregnancy complications reported in AMA pregnancies [65].

Specifically, the trophoblast-specific gene *GNE* showed a synchronous decrease at both the transcriptome and methylome levels in AMA CVS, and the knockdown of *GNE* in the trophoblast cell line HTR8-S/Vneo significantly reduced the migration and invasion ability of cells. *GNE* is a bifunctional enzyme capable of initiating and regulating the biosynthesis of N-acetylneuraminic acid, which is essential for the de novo synthesis of sialic acid [66]. Sialic acid modification of the cell surface plays an important role in cell recognition, cell migration, signal transduction, embryonic development, maternal–fetal interactions, and body aging [66–74]. Homozygous knockouts of *Gne* or *Cmas* (downstream gene of *GNE* in the pathway of biosynthesis of N-acetylneuraminic acid) were lethal to embryos, with significantly decreased or completely eliminated sialic acid modifications on the trophectoderm cell surface [74]. Placental trophectoderm cells are attacked by maternal central complement component 3 (C3), which further leads to embryo loss at E9.5, in a *Cmas* knockout mouse model [74]. In fact, increased DNA methylation levels in CpGs related to *GNE* have been reported in the placenta of IUGR [75]. Thus, the abnormally decreased transcription level due to DNA methylation changes in the DMR near the *GNE* gene might reduce sialic acid modification on the surface of trophoblast cells, resulting in reduced migration and proliferation capabilities as well as maternal–fetal immune tolerance, ultimately leading to an increased risk of SA in AMA pregnancies.

There are also some limitations in our current study. First, the sample size was still small in our study, a larger cohort study would be helpful to verify our findings in future. Second, though RRBS is genome-wide DNA methylation detection technology and the large number of detected CpG sites enables us to explore the AMA’s influence on a greater scale than that using methylation array, it still couldn’t cover all of the CpG sites. Thus, the whole-genome DNA methylation sequencing technologies such as WGBS were recommended in future studies. Third, the application of single-cell transcriptome in the aging field has revealed the great heterogeneity of aging influence among different cell types [76]. Therefore, with the rapid development of single-cell epigenetic assay, we might be able to decipher the epigenetic influences of AMA on different placental cell types at the single-cell level. At last, though the high co-relationships have been observed between the expression of specific DEGs and the DNA methylation level of nearby DMRs, the detailed mechanisms in gene expression regulation need to be further explored for those DMRs.

In conclusion, our study, for the first time, revealed the influence of AMA on DNA methylation at the genome scale, as well as its correlation with transcriptional alterations in early CVS. We uncovered a concealed but close connection between AMA and SA, which provided a new perspective regarding the underlying molecular mechanism of the increased risk of pregnancy complications in AMA.

## Materials and methods

### Participant information

All CVSs were collected from subjects who chose to terminate a pregnancy between 6 and 8 weeks of gestational age (according to the last menstrual period and crown-rump length of the fetus). For the evaluation of AMA’s influence, a total of 24 patients were recruited for the evaluation of AMA’s epigenetic influence and divided into the AMA group (with a mean age of 40.33 ± 1.50, *n* = 12) and the YMA group (mean age, 22.75 ± 2.30, *n* = 12). A total of 10 patients were recruited and divided into the AMA group (with a mean age of 39.25 ± 1.50, *n* = 4) and YMA group (with a mean age of 22.67 ± 2.16, *n* = 6) for the evaluation of the influence of AMA on the transcriptome. The following known strong risk factors for pregnancy loss were excluded: abnormal menstrual cycle, genital infections, antiphospholipid syndrome, and abnormal karyotype in couples. In addition, additional patients with spontaneous abortion were also included in the SA group (mean age, 27.55 ± 2.77, *n* = 11).

### Sample collection and treatment

CVSs were collected immediately after curettage, and maternal tissues were removed using sterilized ophthalmic scissors. Some of the samples were stored in RNAlater (Qiagen, Cat# 76104) at − 20 °C for later RNA extraction, and the rest of the samples were frozen in liquid nitrogen for subsequent DNA extraction.

### DNA and RNA extraction

Genomic DNA (gDNA) was extracted from approximately 20 mg of separated CVS using the QIAamp® DNA mini Kit (Qiagen, Cat# 51306), and total RNA was prepared from 20 to 30 mg of homogenized CVS using the QIAamp® RNA Mini Kit (Qiagen, Cat# 52906), according to the manufacturer's instructions. The gDNA and total RNA were evaluated using a NanoDrop 300 ultraviolet spectrophotometer (ALLSHENG#AS-11020–00). Only samples with an A260/A280 value ranging between 1.8 and 2.0 (for gDNA) and around 2.0 (for total RNA) were used for the experiments.

### Reduced-representation bisulfite sequencing library construction and sequencing

RRBS was performed according to a previously published protocol, with mild modification [77]. In brief, a mixture of 500 ng gDNA and 1 ng λDNA (dam-; dcm-) was digested using FastDigest MspI (Thermo Scientific, Cat# ER0541). The digested DNA was end-repaired and adapter-ligated using the NEBNext Ultra DNA Library Prep Kit (NEB, Cat# E7370) and subsequently digested by Uracil-Specific Excision Reagent (USER) enzyme (NEB, Cat# M5505L) according to the manufacturer’s instructions. The final product was separated by agarose gel electrophoresis (2% TAE gel) and fragments of 200 bp–700 bp DNA were excised and extracted using a gel DNA recovery kit (VISTECH, Cat# DC2005). Bisulfite conversion was conducted using a MethylCode Bisulfite Conversion Kit (Thermo Scientific, Cat# MECOV-50), and PCR amplification of converted DNA was performed with a maximum of 12 cycles with Kapa HiFi U + Master Mix (Kapa Biosystems, Cat# KK2801). Indexes were also introduced during PCR. The libraries were cleaned twice using 0.9X AMPure XP beads (Beckman Coulter, Cat# A63881), and the fragments were qualified using the Fragment Analyzer™ Automated CE System (Analysis Kit: Cat# DNF-474–0500) and a Library Quant Kit for Illumina (NEB Cat# E7630L) and sequenced using the PE150 strategy on an Illumina NovaSeq sequencer.

### Library construction and sequencing of mRNA-seq

mRNA libraries were constructed using the NEBNext® Ultra™ RNA Library Prep Kit for Illumina (NEB Cat# E7770L/E7775L), according to the manufacturer's recommendations. Briefly, poly (A) mRNA was enriched with oligo d(T) beads from approximately 500 ng of total RNA and randomly interrupted with divalent cations in NEB fragmentation buffer. Using fragmented mRNA as the template and random oligonucleotides as primers, the first cDNA strand was synthesized using the M-MuLV reverse transcriptase system; then, the RNA strand was then degraded using RNase H, and the second cDNA strand was synthesized from dNTPs using the DNA polymerase I system. The purified double-stranded cDNA was repaired at the end, added to a tail, and connected to the sequencing connector. Finally, the purified adaptor-ligated DNA fragments were amplified and tagged with specific barcode sequences using PCR. The final mRNA libraries were assessed and sequenced as previously described using the RRBS protocol.

### Data downloading and processing

Single-cell RNA sequencing data of the human placenta [43] was downloaded from https://www.ebi.ac.uk/arrayexpress/experiments/E-MTAB-6701/. We only used the expression information of villous cells for the construction of Seurat objects with the R package Seurat (version 3.2.2) [78], followed by normalization, principal component analysis (PCA), and uniform manifold approximation and projection (UMAP) dimensional reduction with SCTransform, RunPCA, and RunUMAP functions. Subsequently, the cell populations were merged into ten major populations based on their original annotations: cytotrophoblasts (CTBs), syncytiotrophoblasts (STBs), extravillous trophoblasts (EVTs), fetal fibroblasts (fFBs), Hofbauer cells (HB), maternal macrophages (Mycs), endothelial cells (Endo), epithelial cells (Epi), decidual stromal cells (dSTCs), and a mixed population consisting of T cells, B cells, and natural killer cells (TBNKs). The aging and longevity gene lists were downloaded from the Aging Atlas [38] and LongevityMap (Build 3) [39].

### Fundamental data processing for RRBS and mRNA-seq data

Fundamental analysis of the methylome and transcriptome data followed a previous study with slight modifications [77]. Briefly, the raw fastq files were trimmed using TrimGalore software (https://www.bioinformatics.babraham.ac.uk/projects/trim_galore/; version 0.6.6) and Cutadapt (version 1.18) to remove adapters and low-quality bases (Q < 20, length < 36). To evaluate bisulfite conversion rates, the clean reads were first aligned to the phage λ genome using Bismark software (version 0.23.0) [79] with the parameter “bowtie2.” Only samples with bisulfite conversion rates greater than 99% were retained for downstream analysis. Subsequently, Bismark [80] with the parameter “bowtie2” was performed to align clean reads to the Homo sapiens reference genome (human GRCh38/hg38), and only uniquely mapped reads with less than 2% mismatch were retained. Data from both strands were combined and CpGs with more than 5X coverage were retained. To reduce gender bias, CpGs located on sex chromosomes were removed.

For RNA-seq data, the FastQC tool (version 0.11.9) [81] was used to assess data quality. Later, the PCR amplification adapter, poly-N, and low-quality bases (Q < 20, length < 36) were removed from the raw data using TrimGalore software with the parameter of “–quality 20 –stringency 3 –length 36 –paired.” Then, the clean reads were aligned to human GRCh38/hg38 using STAR software (version 2.7.8a) [82] with the default setting and subsequently calculated using the featureCounts function (version 1.6.3) [83] with default settings. Finally, the gene expression level of the normalized count matrix was generated using DESeq2 (version 1.24.0) [84].

### Copy number variation (CNV) analysis

CNV analysis was performed using R package “HMMcopy” (version 1.26.0) [85] at 1 Mb resolution, based on mapped RRBS reads sorted by SAMtools (version 1.3.1) [86]. Using the R function, the “points” and “plot” were applied to plot the CNV.

### DNA methylation level calculation

At each CpG site, the DNA methylation level was estimated as the ratio of the number of reads supporting C (methylated) to the total reads supporting both C and T (methylated and unmethylated). The R package methylKit (version 1.10.0) [87] was used to calculate the DNA methylation level of each retained 200 bp bin that covered more than three CpG sites. The methylation level of each bin was defined as the ratio of the number of reads supporting C (methylated) to the total reads supporting both C and T (methylated and unmethylated) in CpG sites located in these regions. The methylation level of each sample was calculated by averaging the DNA methylation levels of all bins. Next, DNA methylation levels around the genomic region and specific genomic elements were calculated. Specifically, the gene body region from TSS to TES was divided into 100 fractions, while the extended gene body region including -15 kb upstream of the transcription start site (TSS) and 15 kb downstream of the transcription end site (TES) of each gene was split into 100 bp bins to calculate the average methylation level. The DNA methylation levels of each window or fraction were calculated as the mean value of the CpGs within the target region. The average DNA methylation level of each genomic location was then computed to profile the global methylation pattern around the genic region for each sample.

For these special genomic regions, only regions covering no less than three CpG sites were retained, and the DNA methylation level of each region was calculated as the mean value of the methylation level of all CpGs within the target region.

### Principal component analysis (PCA) and clustering analysis

For the RRBS data, the methylation values for these 200 bp bins were used for clustering analysis with the function “clusterSamples” in the R package methylKit (version 1.10.0) [87]and the function “pca” in R package pcaMethods (version 1.10.0) [88].

For the RNA-seq data, the gene expression level calculated by DEseq2 was used for PCA with the R package pcaMethods (version 1.76.0) [88]. The basic R package stats (version 3.6.0) was used to calculate the distance matrices, and the R package heatmap (version 1.0.12) [89] was used for visualization.

### Definition of differentially DNA methylated regions (DMRs)

The DMR analysis only included informative 200 bp tiles covering no less than three CpG sites and existing in eight or more samples per group. Intergroup comparisons between the AMA and YMA groups or between the SA and YMA groups were performed using the R package methylKit (version 1.10.0) [87], and DMRs were defined as 200 bp bins with difference ≥ 15 and q value ≤ 0.05 between these two groups.

### Definition of differentially expressed genes (DEGs)

The intergroup differential expression analysis for the AMA and YMA groups was performed using the R package DESeq2 (version 1.24.1) [84], and AMA-related DEGs were defined as genes with the criteria of adjusted P value less than 0.05, and fold change no less than 1.5 or no more than 0.67.

### Correlation analysis

The average DNA methylation level of each remaining 200 bp bin was calculated for the AMA and YMA groups. The function “stat_cor” in the R package ggpubr (version 0.4.0) [90] was applied to calculate the correlation coefficients and confidence intervals between AMA and YMA groups. Spearman’s correlation coefficients and confidence intervals between maternal age and either the methylation level of selected DMRs or the expression level of selected DEGs were calculated using the functions “cor.test” and “cor” in basic R package stats (version 3.6.0).

### Genomic functional annotation of AMA-related DMRs

The genomic functional annotations for AMA-related DMRs, including the nearest genes, genomic features, and the distance to the transcriptional start site (TSS), were performed using the R package ChIPseeker (version 1.20.0) [31] based on the annotation package TxDb.Hsapiens.UCSC.hg38.knownGene (version 3.10.0) [91]. R package Gviz (version 1.30.3) was applied to present the genomic locations of targeted AMA-related DMRs, the exons of nearby gene, and the covered individual CpG sites [92]. The promoter for specific gene was defined as the region from 3 kb downstream to 3 kb upstream of TSS. The function “foverlaps” in R package data.table (version 1.14.0) [93] was applied to identify DMRs with at least 1-bp overlap with specific genomic elements mentioned in “Data downloading and processing.”

### Gene ontology (GO) and kyoto encyclopedia of genes and genomes (KEGG) enrichment analyses

The “enrichGO” function in R package clusterProfiler (version 3.8.1) [94] was used to identify the enriched GO terms and KEGG pathways for either DEGs or the nearest genes of DMRs.

### Quantitative real-time polymerase chain reaction (qRT-PCR)

The total RNA of CVS in the AMA and YMA groups was reverse-transcribed to cDNA using a reverse transcription kit (Takara, Cat# RR047A). Standard qRT-PCR using SYBR Green PCR Master Mix (Thermo Fisher, Cat# A25742) was carried out with *ACTB* as the endogenous control, and the comparative delta-delta-Ct (^ΔΔ^Ct) method was used to calculate the relative fold difference in gene expression between the AMA and YMA groups. All gene primers used for qRT-PCR are listed below.

*GNE* (F: ACGCAGGGAGCAAAGAG / R: AGCATGGGCAACCAACT)

*GADD45B* (F: ACTGGAGCTGGCGTCTG / R: GTGTGGTCTTGTCGAGGGT)

*ACTB* (F: CATGTACGTTGCTATCCAGGC / R: CTCCTTAATGTCACGCACGAT)

### Cell culture

The human trophoblast HTR8-S/Vneo cell line was purchased from American Type Culture Collection (ATCC, Inc., Manassas, USA), cultured in Roswell Park Memorial Institute (RPMI) 1640 (Gibco, Cat# C11875500BT) containing 10% heat-inactivated fetal bovine serum (FBS; Gibco, Cat# C0235), and then incubated at 37 °C in a 5% CO_2_ incubator.

### Cell transfection

HTR8-S/Vneo cells were plated in 24-well plates and grown to 60% confluence. siRNA targeting *GNE* (si-*GNE*: F:GCUGCCAGAUGUCCUUAAUTT/ R:AUUAAGGACAUCUGGCAGCTT) and negative controls (NC) were transfected into HTR8-S/Vneo cells using RNAi Max (Invitrogen, Cat# 13,778,150). After 48 h of transfection, cells were harvested for subsequent experiments.

### Western blotting

Protein extracts from lysed cells were resolved using 10% sodium dodecyl sulfate–polyacrylamide gel electrophoresis (SDS-PAGE; Thermo Fisher, Cat# NW3010) and transferred to polyvinylidene difluoride membranes (Millipore, Cat# IPVH00010). Membranes were incubated with antibodies against GNE (1:2,000, Abcam, Cat# ab129453) and a monoclonal antibody against β-actin (1:4000, CST, Cat# 2118). Luminescence reagents were added to the blot strips, which were then imaged using an Image Reader LAS-4000 (Fujifilm, Japan).

### Cell proliferation assay

A suspension of 3000 transfected cells in 100 μl culture medium was plated into a 96-well plate. After 12 h of culture in an incubator at 37 °C and 5% CO_2_, the plate was transferred to an IncuCyte® S3 Live-Cell Analysis System (Sartorius, USA). Live cells were monitored over an extended period of time and the data were presented as real-time kinetic data. Each well was scanned every 2 h from 0 to 2 days (48 h). Cell density was calculated using IncuCyte software and phase-contrast images. The data are presented as fold changes in cell density from initiation (0 h) to specific time points during the assay.

### Cell migration assay

The transfected cells were plated in 96-well plates (60,000 cells/well) and cultured overnight. The cellular monolayers were scratched using a 96-pin wound marker, and the medium was changed to serum-free medium according to the manufacturer's instructions (Sartorius, USA). The plate was then transferred to the IncuCyte® S3 Live-Cell Analysis System, and photographs of the plates were taken at 2 h intervals from 0 h to 2 days (48 h). The wound area was calculated at the indicated time points, and the data are presented as the ratio of the area at each time point to that at initiation (0 h) during the assay.

### Cell invasion assay

The transfected cells were plated in 96-well plates (60,000 cells/well) and cultured overnight. Cellular monolayers were scratched using a 96-pin wound marker. After washing away debris using 50 μl of prewarming culture medium, 30 μl of diluted Matrigel (BD Biosciences) (diluted fivefold with culture medium) was added to each well and incubated for 30 min until the Matrigel coagulated. Then, 100 μl of serum-free medium was added to each well according to the manufacturer's instructions (Sartorius, USA). The plate was transferred to the IncuCyte® S3 Live-Cell Analysis System, and photographs of the plates were taken at 2h intervals from 0 h to 2 days (48 h). The wound area was calculated at the indicated time points, and the data are presented as the ratio of the area at each time point to that at initiation (0 h) during the assay.

### Statistical analysis

The unpaired two-tailed t test of GraphPad Prism Version 9.4.0 (GraphPad Software, Inc., San Diego, CA, USA) was used to determine the significance of intergroup differences in gene expression measured by qRT-PCR. The Wilcoxon rank-sum test in the function "stat_compare_means" of R package ggpubr (Version 0.4.0) [90] was used to determine the significance of differences between the AMA and YMA groups for the DNA methylation level of the target region and the expression level of the selected gene. Spearman-based correlation tests were used to determine the correlation coefficients and P values for the DNA methylation pattern between the AMA and YMA groups, between the DNA methylation level of special DMR and maternal age, and between the expression level of special genes and maternal age (ns: *P* ≥ 0.05;  *P < 0.05;  **P < 0.01;  ***P < 0.001).

## Supplementary Information

**Additional file 1. Figure. S1.** Basic data quality evaluation for each reduced-representation bisulfite (RRBS) library.

**Additional file 2. Figure. S2.** Overview of DNA methylation patterns for all samples.

**Additional file 3.** **Figure. S3.** Indentation and features of AMA-related DMRs.

**Additional file 4. Figure. S4.** Quality evaluation and the profiling of transcriptome data.

**Additional file 5.** **Figure. S5.** Detail information of AMA-related DMRs near CDK11A and C19ORF71 genes.

**Additional file 6.** **Figure. S6.** Detail information of AMA-related DMRs near COL5A1 and GNE genes.

**Additional file 7.** **Figure. S7.** Genomic location information of 21 AMA-related DMRs.

**Additional file 8.** **Figure. S8.** Correlation analysis for targeted AMA-related DEGs and DMRs.

**Additional file 9.** **Figure. S9.** Quality evaluation of RRBS data in the SA group.

**Additional file 10.** **Figure. S10.** Overview of the DNA methylation patterns of DMRs near 25 overlapping genes among all samples in the YMA, AMA and SA groups.

**Additional file 11.** **Figure. S11.** The expression pattern of 25 overlapping genes in single-cell villous altas.

**Additional file 12.** **Figure. S12.** Proliferation and invasion assays for the GNE–siRNA-inhibited trophoblast cell line.

**Additional file 13.** **Table S1. **Clinical features of samples in the RRBS and mRNA-seq.

**Additional file 14.** **Table S2. **Summary of the sequencing information of all RNA-seq and RRBS libraries, as well as the number and average coverage depth of CpGs detected at different minimum coverage values for each RRBS library.

**Additional file 15.** **Table S3. **List of AMA-related DMRs. DMRs:200bp bins with |differential DNA methylation value| > 15% and q value < 0.05.

**Additional file 16.** **Table S4.** Gene Ontology (biological process) enrichment terms and KEGG pathways for the nearest genes to AMA-related DMRs.

**Additional file 17.** **Table S5. **List of AMA-related DEGs. DEGs: gene with adjusted P value < 0.05, and |log2 fold change| ≥ 0.585.

**Additional file 18.** **Table S6.** Representative enrichment of Gene Ontology (biological process) terms and KEGG pathways for AMA-related DEGs.

**Additional file 19.** **Table S7. **Genomic annotation information for DMRs whose nearby genes belong to AMA-related DEGs (n=25).

**Additional file 20.** **Table S8. **List of SA-related DMRs. DMRs:200 bp bins with |differential DNA methylation value| > 15% and q value < 0.05.

## Data Availability

The sequencing datasets in this study have been deposited in the National Genomics Data Center database (accession number: HRA001875) upon request from any qualified researcher who meets the criteria for access to confidential data.
