# Supplementary figures and images for: DNA methylation abnormalities induced by advanced maternal age in villi prime a high-risk state for spontaneous abortion
Source: Clin Epigenetics. 2023 Mar 21;15:44. doi: 10.1186/s13148-023-01432-w (PMC10029192; doi:10.1186/s13148-023-01432-w)

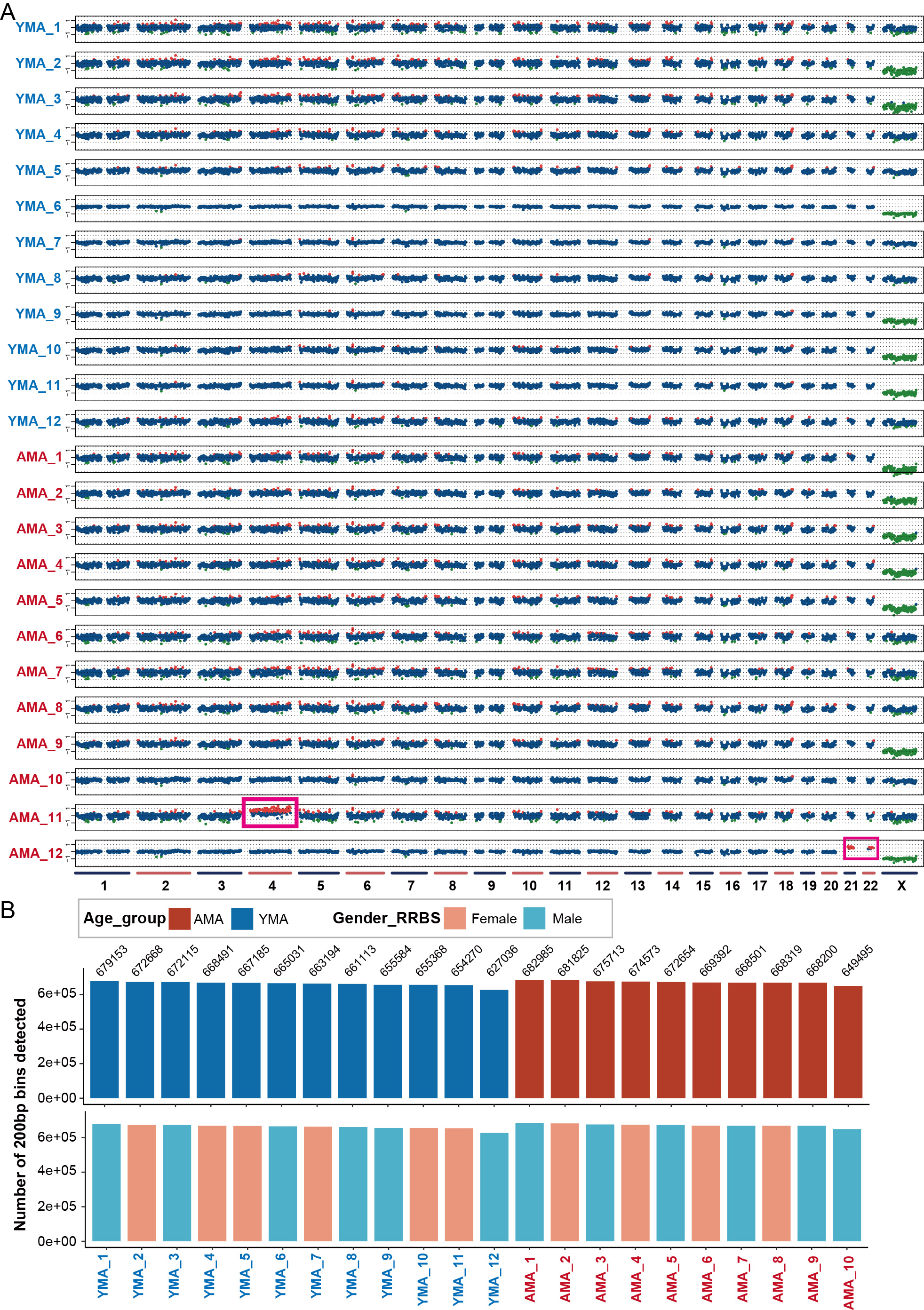

Supplement: Supplementary file 1 — Additional file 1. Figure. S1. Basic data quality evaluation for each reduced-representation bisulfite (RRBS) library. [file 13148_2023_1432_MOESM1_ESM.jpg]

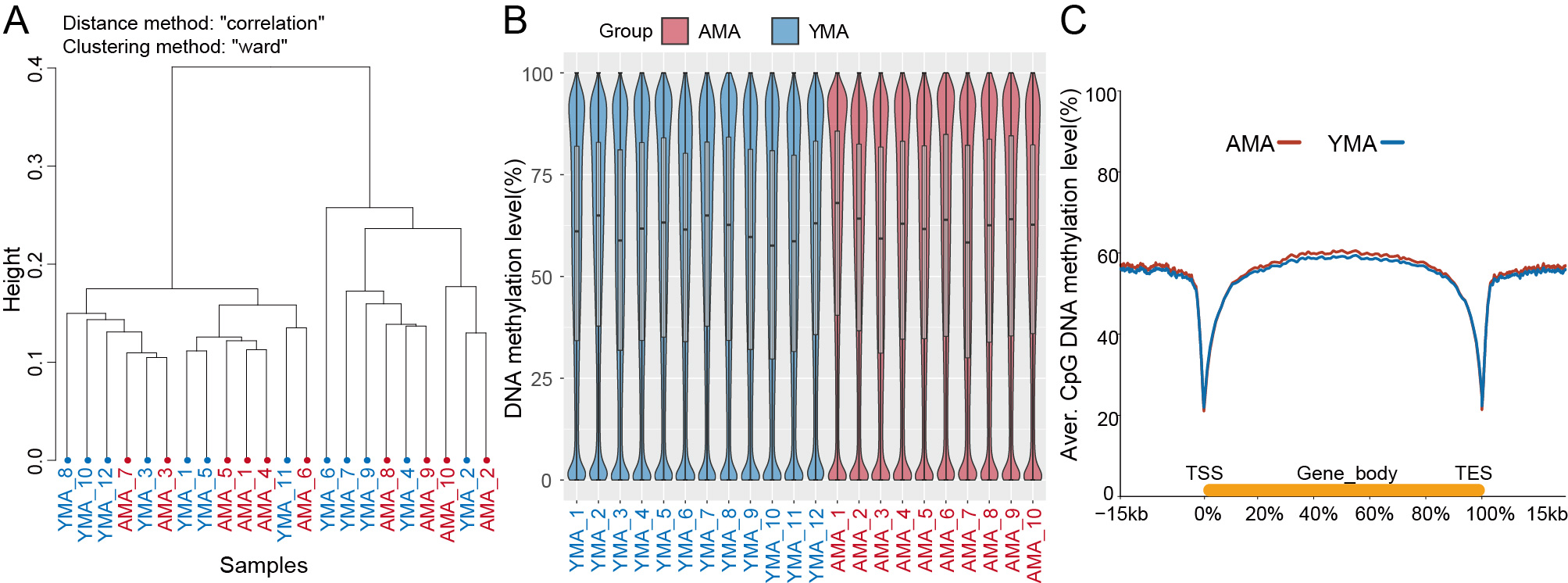

Supplement: Supplementary file 2 — Additional file 2. Figure. S2. Overview of DNA methylation patterns for all samples. [file 13148_2023_1432_MOESM2_ESM.jpg]

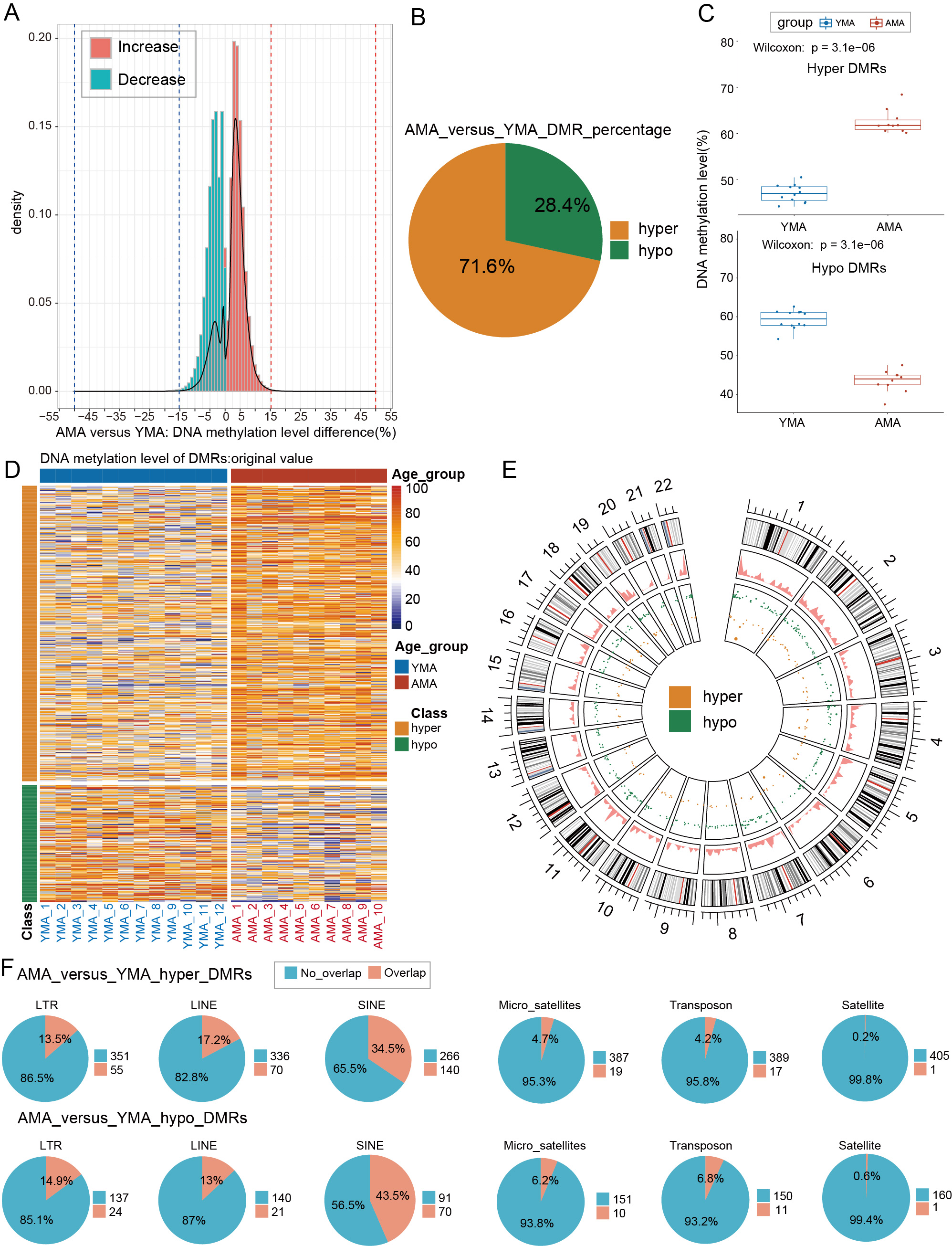

Supplement: Supplementary file 3 — Additional file 3. Figure. S3. Indentation and features of AMA-related DMRs. [file 13148_2023_1432_MOESM3_ESM.jpg]

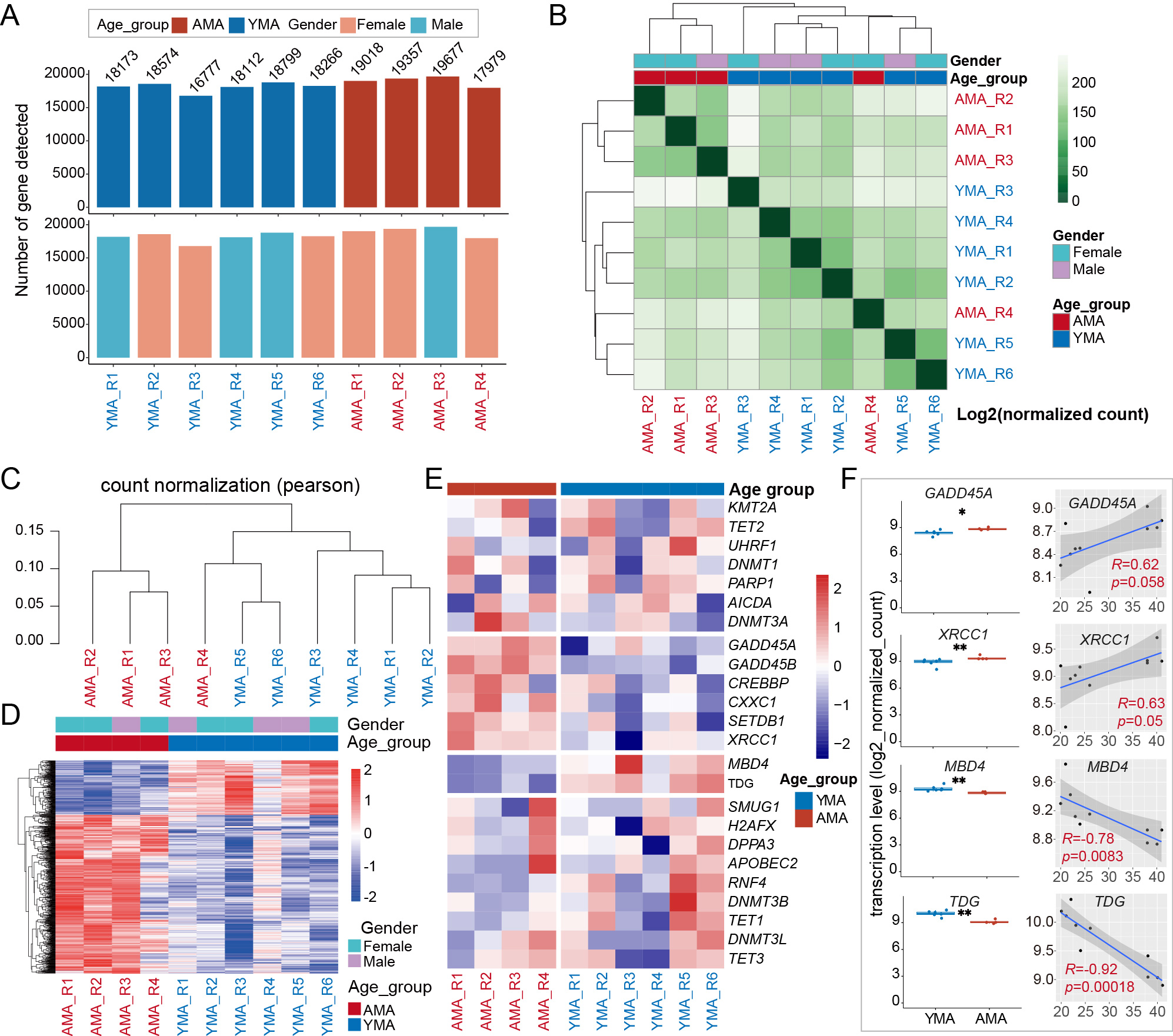

Supplement: Supplementary file 4 — Additional file 4. Figure. S4. Quality evaluation and the profiling of transcriptome data. [file 13148_2023_1432_MOESM4_ESM.jpg]

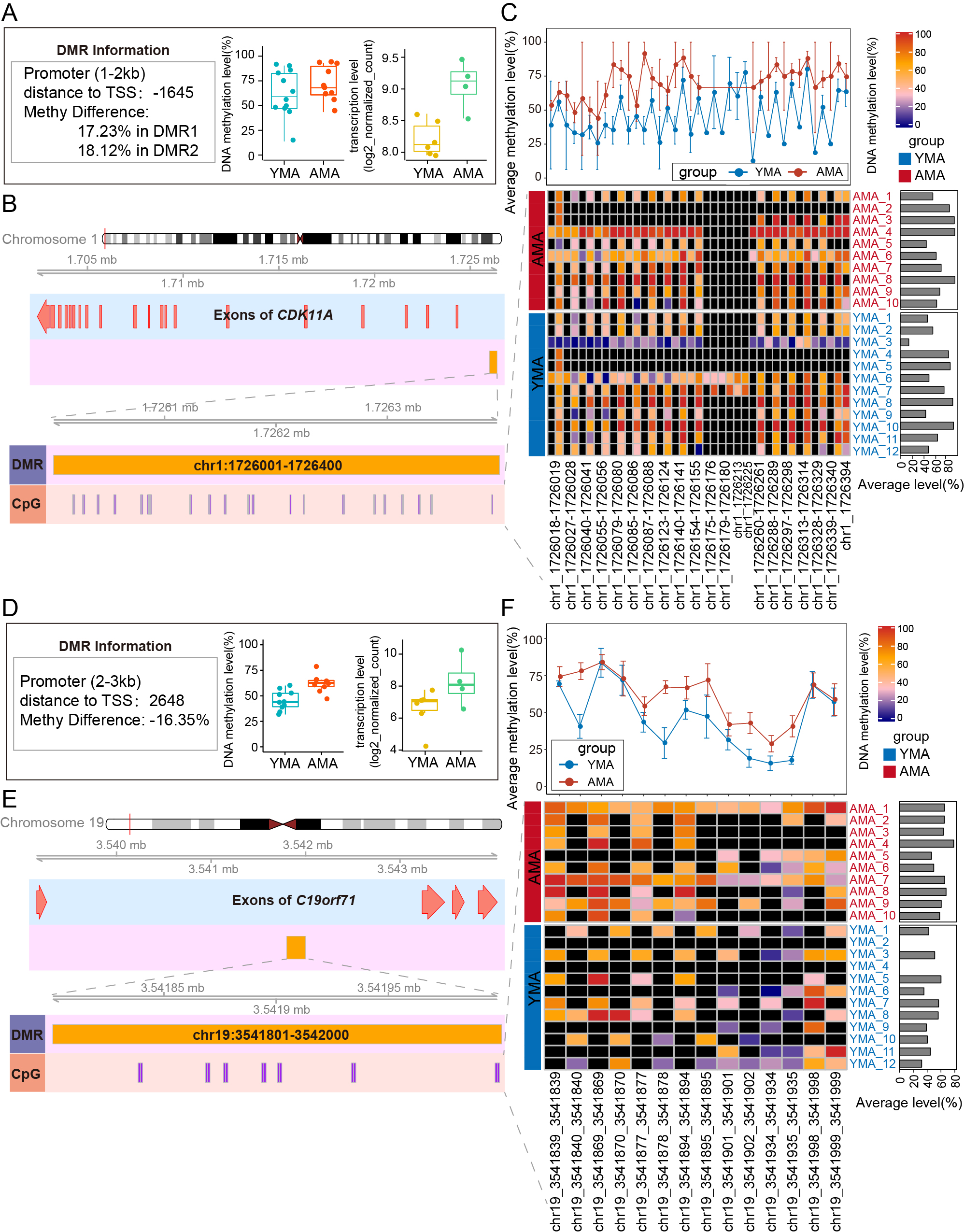

Supplement: Supplementary file 5 — Additional file 5. Figure. S5. Detail information of AMA-related DMRs near CDK11A and C19ORF71 genes. [file 13148_2023_1432_MOESM5_ESM.jpg]

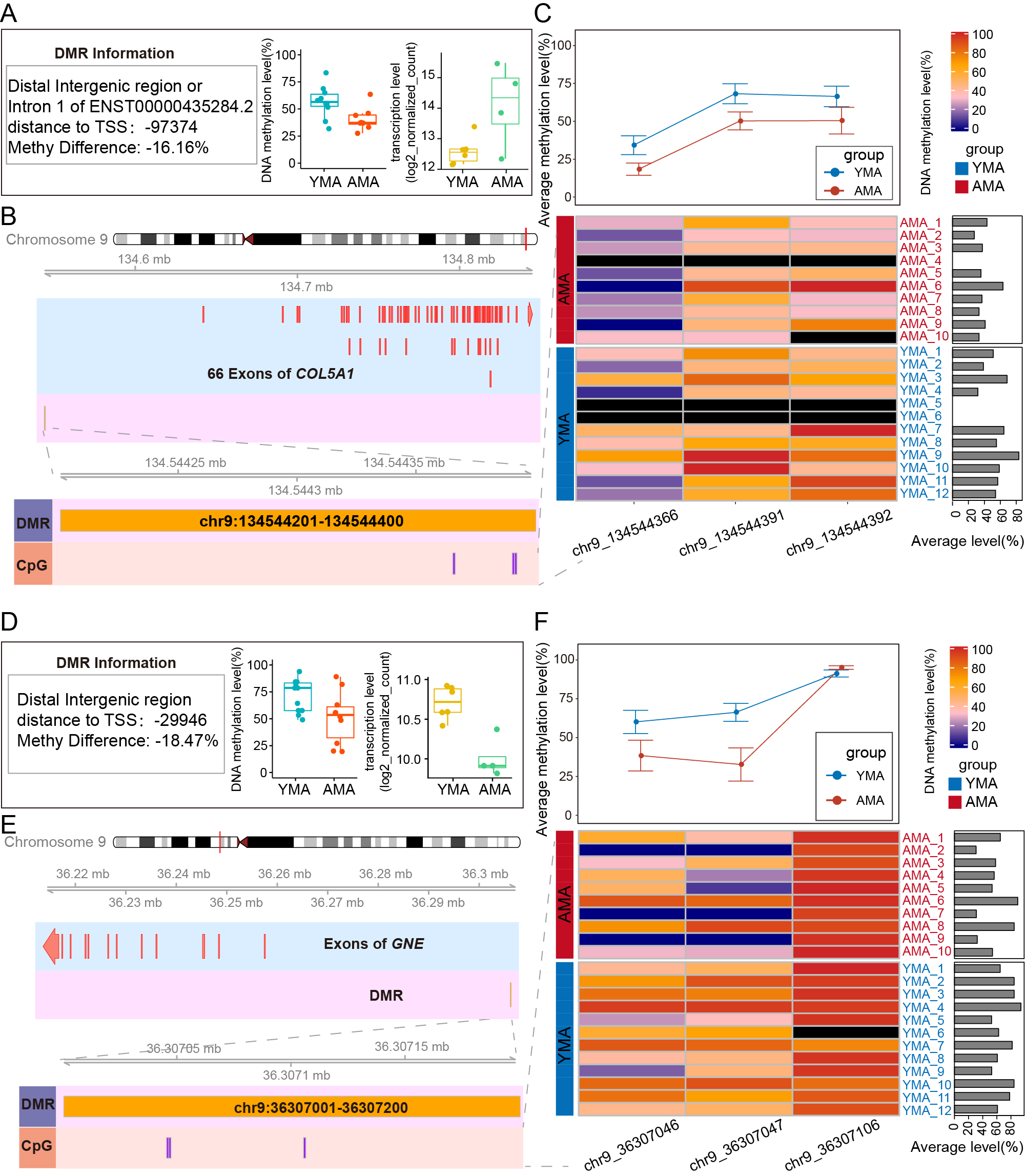

Supplement: Supplementary file 6 — Additional file 6. Figure. S6. Detail information of AMA-related DMRs near COL5A1 and GNE genes. [file 13148_2023_1432_MOESM6_ESM.jpg]

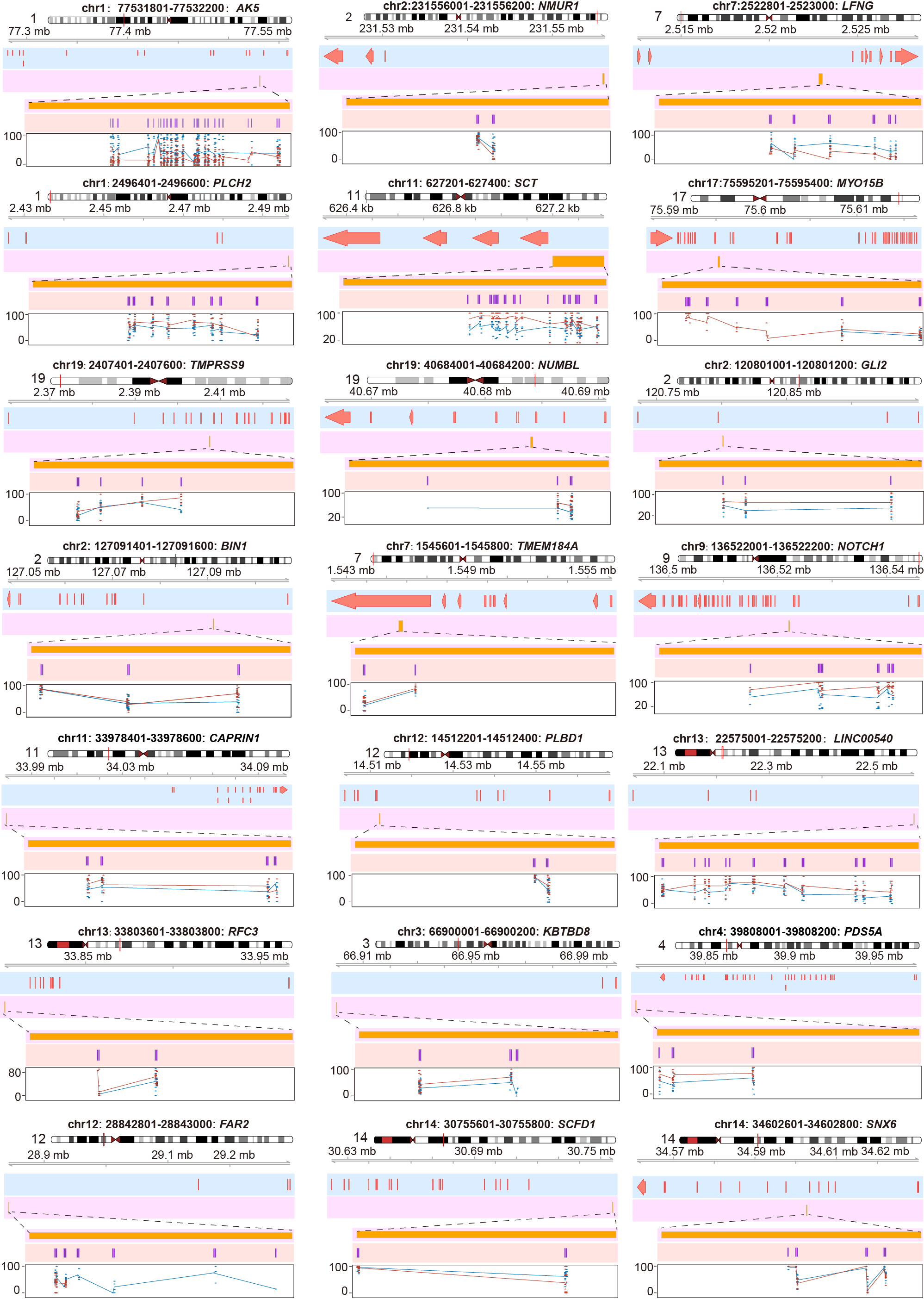

Supplement: Supplementary file 7 — Additional file 7. Figure. S7. Genomic location information of 21 AMA-related DMRs. [file 13148_2023_1432_MOESM7_ESM.jpg]

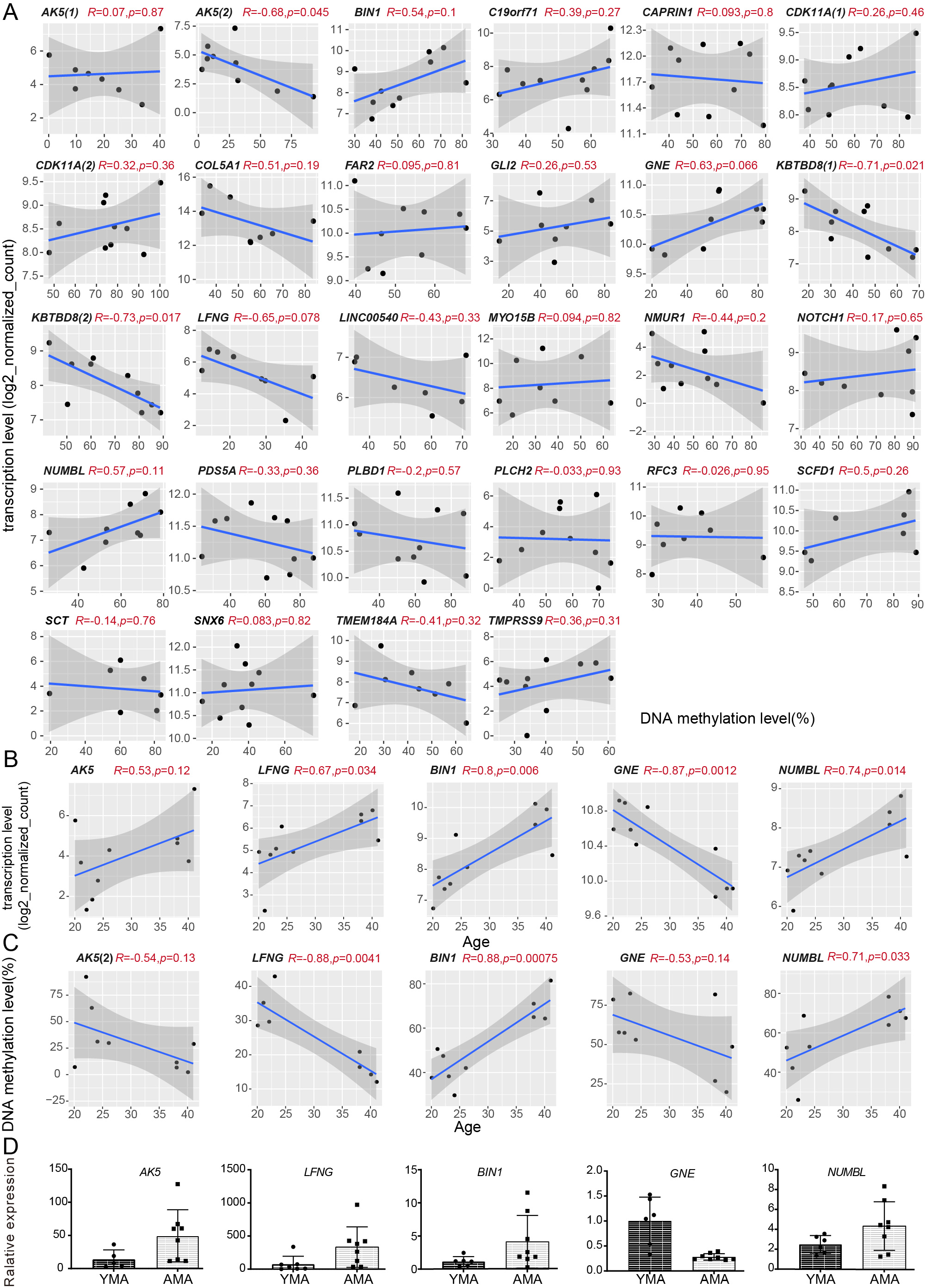

Supplement: Supplementary file 8 — Additional file 8. Figure. S8. Correlation analysis for targeted AMA-related DEGs and DMRs. [file 13148_2023_1432_MOESM8_ESM.jpg]

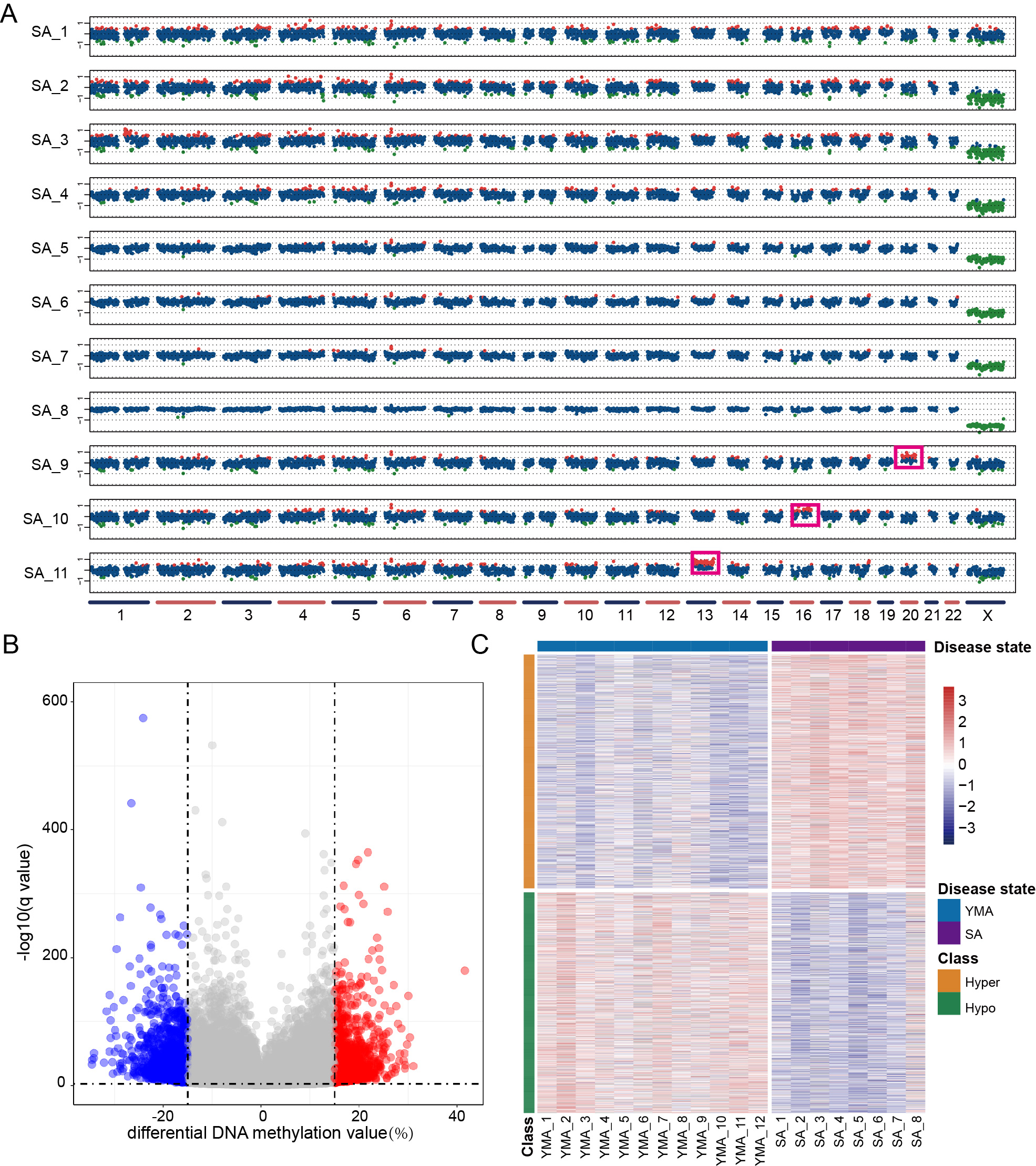

Supplement: Supplementary file 9 — Additional file 9. Figure. S9. Quality evaluation of RRBS data in the SA group. [file 13148_2023_1432_MOESM9_ESM.jpg]

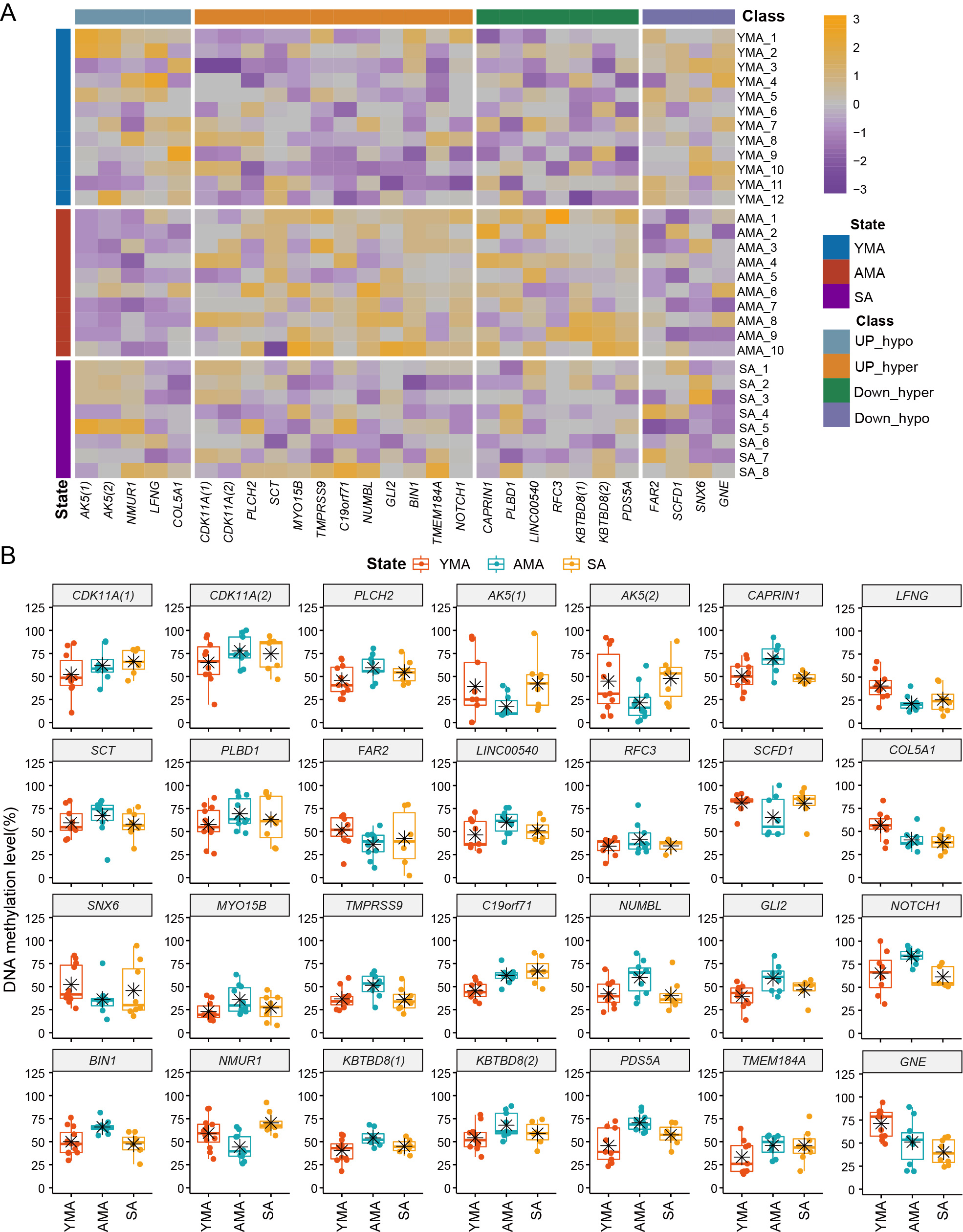

Supplement: Supplementary file 10 — Additional file 10. Figure. S10. Overview of the DNA methylation patterns of DMRs near 25 overlapping genes among all samples in the YMA, AMA and SA groups. [file 13148_2023_1432_MOESM10_ESM.jpg]

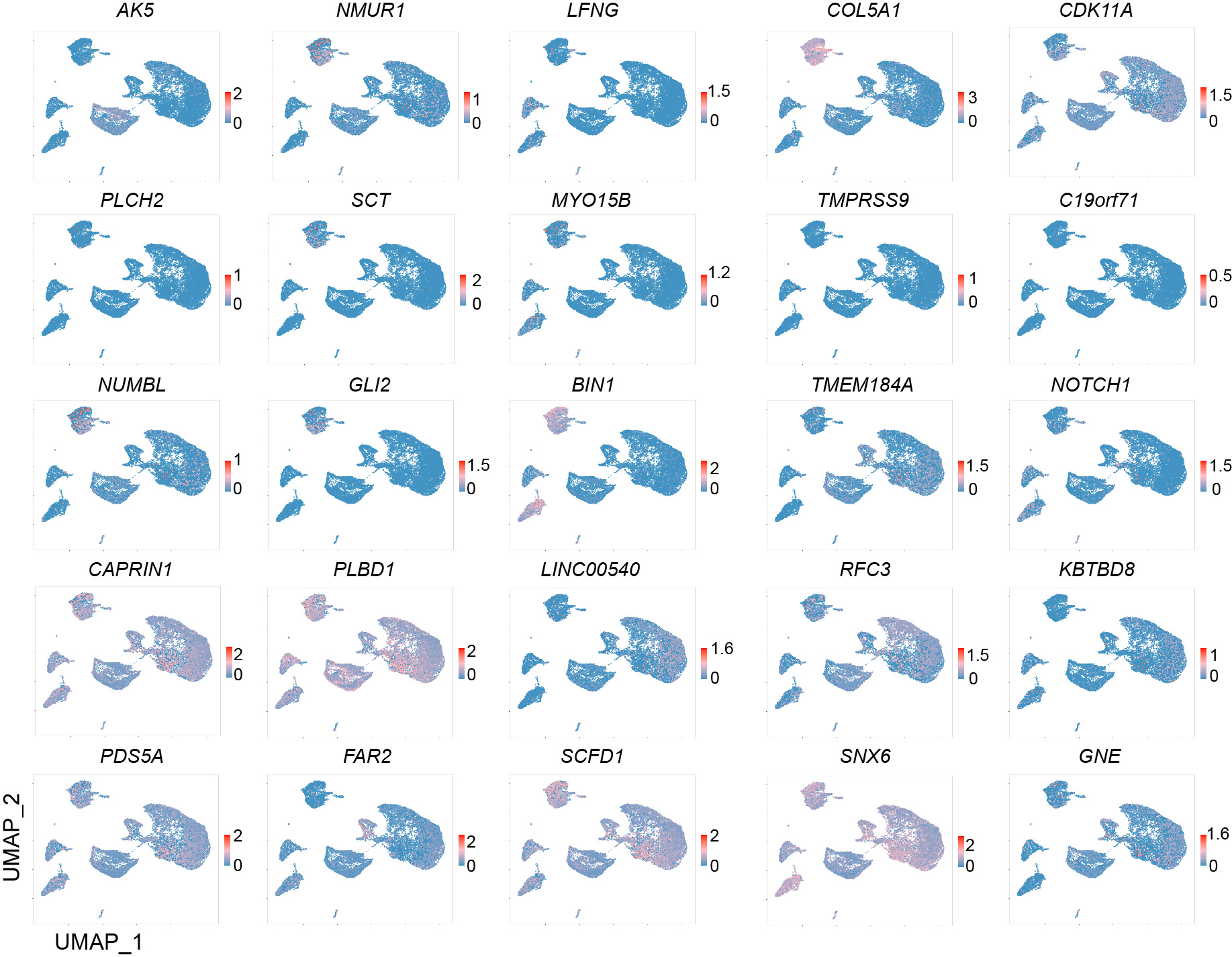

Supplement: Supplementary file 11 — Additional file 11. Figure. S11. The expression pattern of 25 overlapping genes in single-cell villous altas. [file 13148_2023_1432_MOESM11_ESM.jpg]

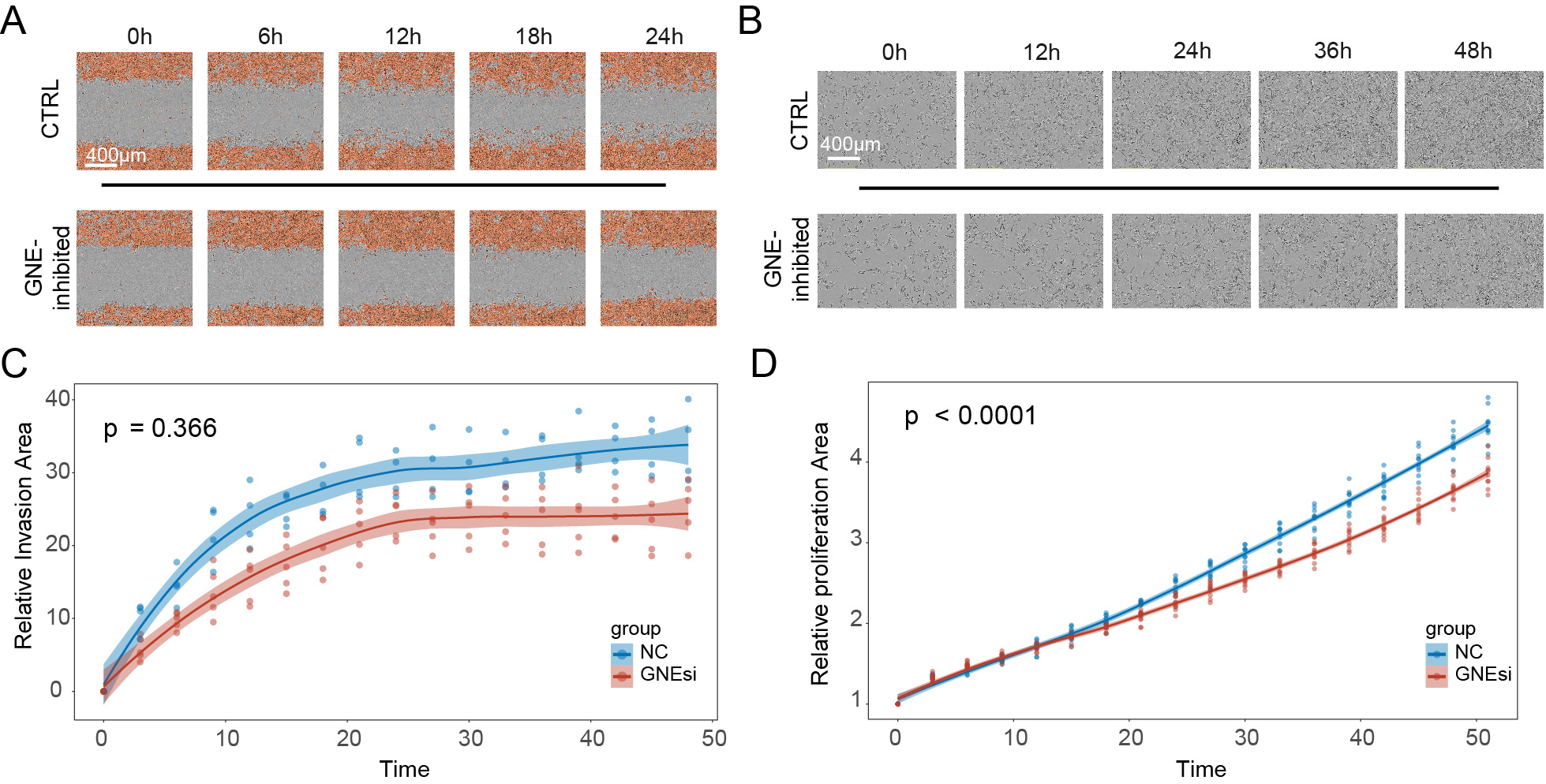

Supplement: Supplementary file 12 — Additional file 12. Figure. S12. Proliferation and invasion assays for the GNE–siRNA-inhibited trophoblast cell line. [file 13148_2023_1432_MOESM12_ESM.jpg]
